# The relative abundances of yeasts attractive to *Drosophila suzukii* differ between fruit types and are greatest on raspberries

**DOI:** 10.1038/s41598-022-14275-x

**Published:** 2022-06-20

**Authors:** Rory Jones, Michelle T. Fountain, Nadia A. Andreani, Catrin S. Günther, Matthew R. Goddard

**Affiliations:** 1 School of Life Sciences, University of Lincoln, Lincoln, LN6 7DL UK; 2 NIAB EMR, New Road, East Malling, Kent, ME19 6BJ UK; 3 The School of Biological Sciences, The University of Auckland, Auckland, New Zealand; 4 Present Address: Max Planck Institute for Evolutionary Biology, Plön, Germany; 5 Present Address: The New Zealand Institute of Plant and Food Research Ltd, Ruakura Research Campus, Bisley Road, Hamilton, 3214 New Zealand

**Keywords:** Microbial ecology, Ecology, Fungi, Fungal ecology, Microbial ecology, Microbiology, Environmental microbiology, Food microbiology

## Abstract

Fungal metabolic volatiles attract *Drosophila suzukii* which oviposits in ripening fruits, but there are few data describing the fungal microbiomes of commercial fruits susceptible to this insect pest. We tested the hypothesis that fruit type and ripening stage have a significant effect on fruit surface fungal communities using DNA metabarcoding approaches and found strong support for differences in all three fungal community biodiversity metrics analysed (numbers, types, and abundances of taxa). There was an average fivefold greater difference in fungal communities between sites with different fruit types (strawberry, cherry, raspberry, and blueberry) than across fruit developmental stages, demonstrating site and/or fruit type is the greater factor defining fungal community assemblage. The addition of a fungal internal standard (*Plectosphaerella cucumerina*) showed cherry had relatively static fungal populations across ripening. Raspberry had a greater prevalence of Saccharomycetales yeasts attractive to *D. suzukii*, including *Hanseniaspora uvarum,* which aligns with reports that raspberry is among the fruits with greatest susceptibility and attraction to *D. suzukii*. Greater knowledge of how yeast communities change during fruit maturation and between species or sites may be valuable for developing methods to manipulate fruit microbiomes for use in integrated pest management strategies to control *D. suzukii*.

## Introduction

Fungi are widespread in the environment and are important components of agricultural and natural ecosystems where they play key roles in nutrient turnover. Fruit associated fungi may have both positive and negative impacts on the quality of products derived from horticultural systems by causing spoilage^1^ or beneficial attributes in fermented beverages such as wine^2^. Fruit surfaces are home to complex and dynamic microbial communities which are affected by a number of factors including fruit species^3,4^ and variety^5,6^, ripening stage^7–9^, plant organ^9^, geographic location^6,10–12^ and farming practices^13,14^*.* Fruit fungal communities are dominated by the Ascomycota and Basidiomycota phyla, with Ascomycota comprising 52–97% and Basidiomycota 4–24% of species on a range of fruits including *Vitis vinifera* (grape)^12^, *Malus pumila* Mill. (apples)*, Ribes nigrum* (blackcurrants)^4^, and *Fragaria* × *ananassa* (strawberries)^9^.

*Drosophila* are saprotrophic and thus dependent on microbes for nutrition; complex interactions between *Drosophila*, microbes and fruit have been described^15^. Unlike most other *Drosophila* species, *Drosophila suzukii* is able to oviposit in ripening fruit due to a morphologically modified ovipositor^16^. *Drosophila suzukii* causes economic losses through direct fruit damage by ovipositing and subsequent larval feeding, including indirect damage caused by secondary infection from microbes via wounds as entry points^17–19^. The economic damage caused by *D. suzukii* is significant, with losses estimated at $511.3 million in just three USA states in 2008^20^. Recent geographic range expansion including invasions into the USA and mainland Europe in 2008 and the UK in 2012^20–22^ have resulted in *D. suzukii* now significantly threatening soft and stone fruit production in most Northern Hemisphere temperate regions. Yeasts are an important source of nutrients for *D. suzukii* as they provide a protein source important for egg development^23^. Female *D. suzukii* prefer to oviposit on yeast-colonised fruits^24^, and female fecundity, larva development and survival is affected by yeast species^25,26^. It is increasingly documented that *D. suzukii* are attracted to volatile chemicals produced by budding yeasts in the Saccharomycetales order, including *Hanseniaspora uvarum*, *Hanseniaspora opuntiae*, *Saccharomyces cerevisiae*, *Metschnikowia pulcherrima*, *Candida zemplinina*, *Candida californica, Pichia terricola* and *Pichia pijperi*^27–31^. In addition to single species, we have recently shown that various combinations of yeasts involving *C. zemplinina*, *P. pijperi*, *M. pulcherrima* and *H. uvarum* are attractive to *D. suzukii*^30^. Moreover, there is an overlap between Saccharomycetales yeasts found on cherry and raspberry and those in *D. suzukii* guts, particularly *Hanseniaspora* species^32,33^. There is also recent evidence to show reduced olfactory attraction to raspberries when they are infected with *Botrytis cinerea*, a fruit fungal pathogen^34^. These observations can be exploited for the control of *D. suzukii* in various traps and baits^15^. Thus, there is value in understanding the general communities of fungi and especially the communities of Saccharomycetales naturally associated with various fruits as they ripen as these may modulate the attraction of *D. suzukii.*

Fungi associated with crops and foods were originally evaluated by culture-based approaches, but work shows that up to 95% of fungi on fruits may be missed using these methods^12^. The PCR amplification of specific diagnostic ‘barcode’ areas from DNA which has been directly extracted from substrates of interest may circumvent this non-culturable issue^12^. DNA barcode metagenomics studies have reported significant differences between fungal communities on the surfaces of apples and blackcurrants^4^, as well as between *Hippophae rhamnoides* L. (sea buckthorn), *Aronia melanocarpa* (black chokeberry), and *Ribes rubrum* (red and white currants)^3^. Further, there are reports that fungal communities differ between varieties within fruit species in both the Northern and Southern Hemisphere, e.g. between Chardonnay and Syrah grape varieties in New Zealand^6^ and Zinfandel, Chardonnay and Cabernet Sauvignon in California^10^. One of the drawbacks of barcode amplicon sequencing (and metagenomic analyses generally) is that it only allows the relative abundances of taxa to be analysed, and the underlying absolute biological abundances of taxa are not known unless controls for abundances are included. Various metagenomic studies have attempted to quantify absolute abundances by the use of internal standards^35^, but to our knowledge no previous work has attempted this for fungal communities on fruit. In addition, there are few data characterising changes in fruit fungal communities through ripening generally. Studies on table grapes and sea buckthorn berries suggested these microbial communities changed over time^8,9,36^. Abdelfattah et al.^9^ observed significant differences in fungal community structure between immature and mature strawberry fruit with unweighted UniFrac analysis (*P* = 0.003), but communities were dominated by *Botrytis* and *Cladosporium* genera, suggesting the difference across ripening was driven by a subtle shift in rare taxa.

Data on the microbial composition of fruits and their maturation-related changes is generally limited. We are not aware of any studies that have comprehensively identified the fungal and yeast communities associated with different ripening stages of commercially important fruit which are susceptible to *D. suzukii*. To help fill this knowledge gap, we investigated the general fungal and Saccharomycetales (budding yeasts) communities on blueberry, cherry, raspberry, and strawberry during ripening in the UK, using a barcode metagenomics approach. Further, we aimed to do this quantitatively by spiking samples with a known number of *Plectosphaerella cucumerina* cells as an internal standard. We test the hypothesis that both fruit type and ripening stage have a significant effect on general fungal and Saccharomycetales yeast communities and evaluate whether there are differences in specific yeasts known to be attractive to *D. suzukii*.

## Results

Six biological replicates each were sampled from four fruit species (blueberries, cherries, raspberries, and strawberries) at four developmental stages. Developmental stages were based on fruit pigmentation ranging from unripe (green) to fully ripe (red/purple/navy; Fig. S1) throughout June to September in 2018. Ten fruits (except blueberries N = 20) were collected for each species per replicate, and this was replicated six times for each ripening stage for each fruit at different sites.

### Quantitative analysis of fungal communities

**Figure 1:**
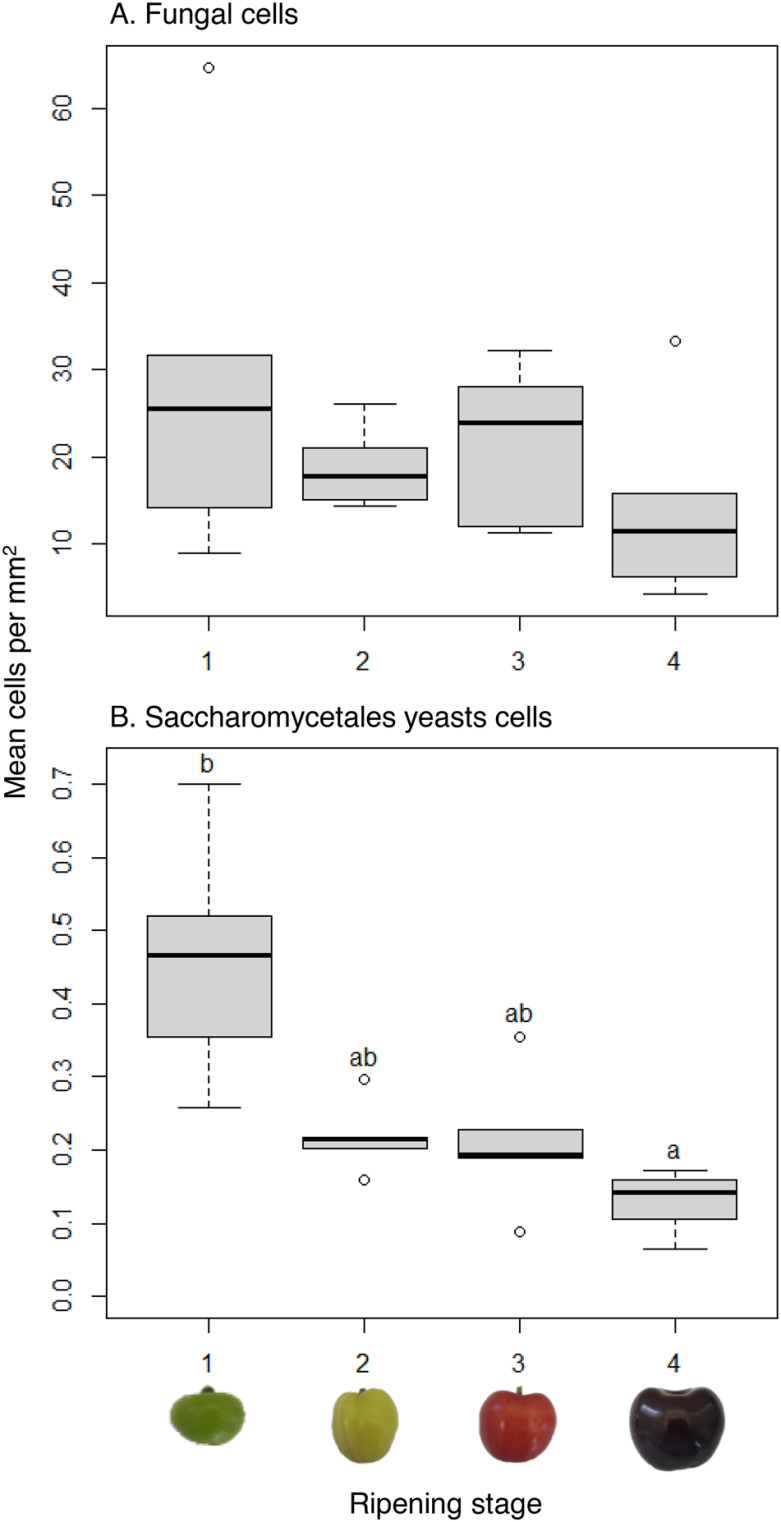
Absolute fungal cell abundances on cherry epicarp. Number of total fungal (**A**) and Saccharomycetales yeasts (**B**) cells per mm^2^ of cherry epicarp (N = 6 except, stage 3 and 4, N = 5) at four ripening stages (1, unripe/green fruit; 2, de-greening fruit; 3, ripening fruit; and 4, fully ripe/harvest fruit) estimated from DNA read abundances normalised to DNA abundances from the deliberate addition of 265 live *Plectosphaerella cucumerina* cells prior to DNA extraction. Different lower-case letters above bars show significant differences between ripening stages at *P* > 0.05, Dunn’s comparisons post-hoc with Benjamini–Hochberg multiple comparison correction.

Metabarcoding analysis is generally not quantitative, but the addition of 265 *P. cucumerina* cells to sub-samples prior to DNA extraction served as an internal standard to attempt an estimation of the size of fungal populations. One replicate spiked with the internal standard of the strawberry stage 3 samples was removed due to poor sequence quality leaving 96 non-spiked and 95 spiked samples which produced a total of 38,445,395 reads that clustered into 1712 > 97% identity Amplicon Sequence Variants (ASV), which from here-in we call phylotypes (Table S1). Blast searches across all phylotypes for matches to the *P. cucumerina* internal standard’s ITS sequence generated from Sanger sequencing revealed one phylotype that matched with 100% identity. *Plectosphaerella cucumerina* was naturally present in 21 of the 95 non-spiked samples and comprised of a total of 444 reads. Cherry was the only fruit where the internal standard was reliably recovered: 23 of 24 spiked samples and only one of 24 non-spiked samples contained the internal standard phylotype. After internal standard DNA read normalisation, the mean (± SE) number of fungal cells from each of the useable 23 pairs of cherry replicates was 307,323 (± 39,090) cells. The range of phylotype cell abundance across all cherry samples was 3.9 million for an *Aureobasidium* phylotype to 3 cells for a phylotype taxonomically assigned no higher level than kingdom. There was no significant change in total fungal cell numbers across cherry maturation stage (Kruskal–Wallis, chi-squared = 2.63, *P* = 0.45; Fig. S2), but fruit surface areas also increased significantly (Kruskal–Wallis, chi-squared = 19.70, *P* = 0.0002, Fig. S2). When cell numbers were normalised for surface area this revealed that absolute fungal population sizes remained static across cherry maturation stages (Kruskal–Wallis, chi-squared = 2.49, *P* = 0.48; Fig. 1A). However, there was a significant change in absolute Saccharomycetales cell numbers when normalised for cherry surface area across maturation (Kruskal–Wallis, chi-squared = 15.30, *P* = 0.002): stage 1 had significantly greater absolute Saccharomycetales cell numbers than stage 4 (*P* = 0.0007; Fig. 1B). Six individual Saccharomycetales yeast phylotypes from the genera *Debaryomyces*, *Saccharomyces*, *Kodamaea*, one from the family Pichiaceae, and phylotypes with > 97% homology to *M. pulcherrima* and *Metschnikowia gruessii*, had significantly greater abundances on ripening stage 1 than 4 (*P* values span 0.045 to 0.006).

### Overview of fungal diversity across all fruit samples

The *P. cucumerina* internal standard phylotype was removed from all samples, and the sequence data normalised and analysed. A total of 1712 fungal phylotypes was revealed, comprising seven phyla, 25 classes, 96 orders, 197 families, and 280 genera. The most abundant and diverse phylum was Ascomycota, comprising 92.2% of reads and 57.3% of phylotypes, followed by Basidiomycota (7.7% reads and 33.6% phylotypes), Zygomycota (0.1% and 1.1%), Chytridiomycota (> 0.1% and 0.7%), Mucoromycota (> 0.1% and 0.3%), Glomeromycota and Rozellomycota (both > 0.1% and 0.1%; Fig. S3A). A phylotype from the *Cladosporium* genus was the most common phylotype across all samples, comprising 60.8% of reads. A total of 87 phylotypes from the order Saccharomycetales (budding yeasts) was detected, comprising 1,792,782 DNA reads (4.7% of the total) spanning 10 families and 25 genera. *Metschnikowia* was the most abundant Saccharomycetales genus (40.0% of Saccharomycetales reads), followed by *Hanseniaspora* (38.2%), then *Pichia* (5.2%), with the remaining genera contributing fewer than 3% each. *Candida* was the most diverse genus within the order Saccharomycetales accounting for 21.8% of phylotypes, despite only comprising 2.4% of reads, followed by *Metschnikowia* (11.5%), *Hanseniaspora* (8.0%) and *Pichia* (6.9%), with each of the remaining genera contributing fewer than 3.5% of phylotypes each (Fig. S3B). The most common Saccharomycetales yeast across all samples was a phylotype from the genus *Hanseniaspora* with > 97% homology to *H. uvarum* and comprised 38.2% of the total Saccharomycetales reads (Fig. S3B).

### The effect of fruit species and ripening stage on epicarp fungal communities

We analysed differences in three biodiversity metrics to evaluate the effect of fruit species and maturation stage on fungal communities: differences in the absolute numbers of phylotypes (richness); differences in the types of phylotypes (i.e. presences/absences); and differences in the relative abundances of phylotypes (community composition) following Morrison-Whittle et al.^14^ and Morrison‐Whittle and Goddard^37^.

#### Fungal phylotype richness

Phylotype richness was not normally distributed (Shapiro-Wilks, *P* = 0.008) but square root transformation allowed the data to conform to the assumptions of ANOVA. There was a significant effect of both fruit type and ripening stage on the number of fungal phylotypes, including an interaction between the two (*F*_3,175_ = 18.58, *P* = 1.65 × 10^–10^; *F*_3,175_ = 5.00, *P* = 0.002 and *F*_9,175_ = 6.69, *P* = 3.25 × 10^–8^ respectively). Comparisons of effect sizes revealed fruit type (ω^2^ = 0.30) had a 4.4 times greater effect than ripening stage (ω^2^ = 0.068) on fungal phylotype richness. Disregarding ripening stage, cherry (mean ± SE number of phylotypes = 98 ± 4.1) had significantly more fungal phylotypes than blueberry (68 ± 3.7), raspberry (72 ± 2.9) and strawberry (76 ± 3.2) (Tukey’s HSD, *P* < 1.0 × 10^–7^, *P* = 2.0 × 10^–7^ and *P* = 2.56 × 10^–5^ respectively), which did not differ from one another (Fig. S4). Disregarding fruit type, ripening stage 2 (mean ± SE number of phylotypes = 85 ± 2.9) and 3 (82 ± 4.1) had significantly more fungal phylotypes than stage 1 (*P* = 0.001 and *P* = 0.033, respectively), but numbers at stages 1 and 4 were not significantly different (Fig. S4). The absolute time points for sampling did however differ between fruits due to different maturation timings.

**Figure 2:**
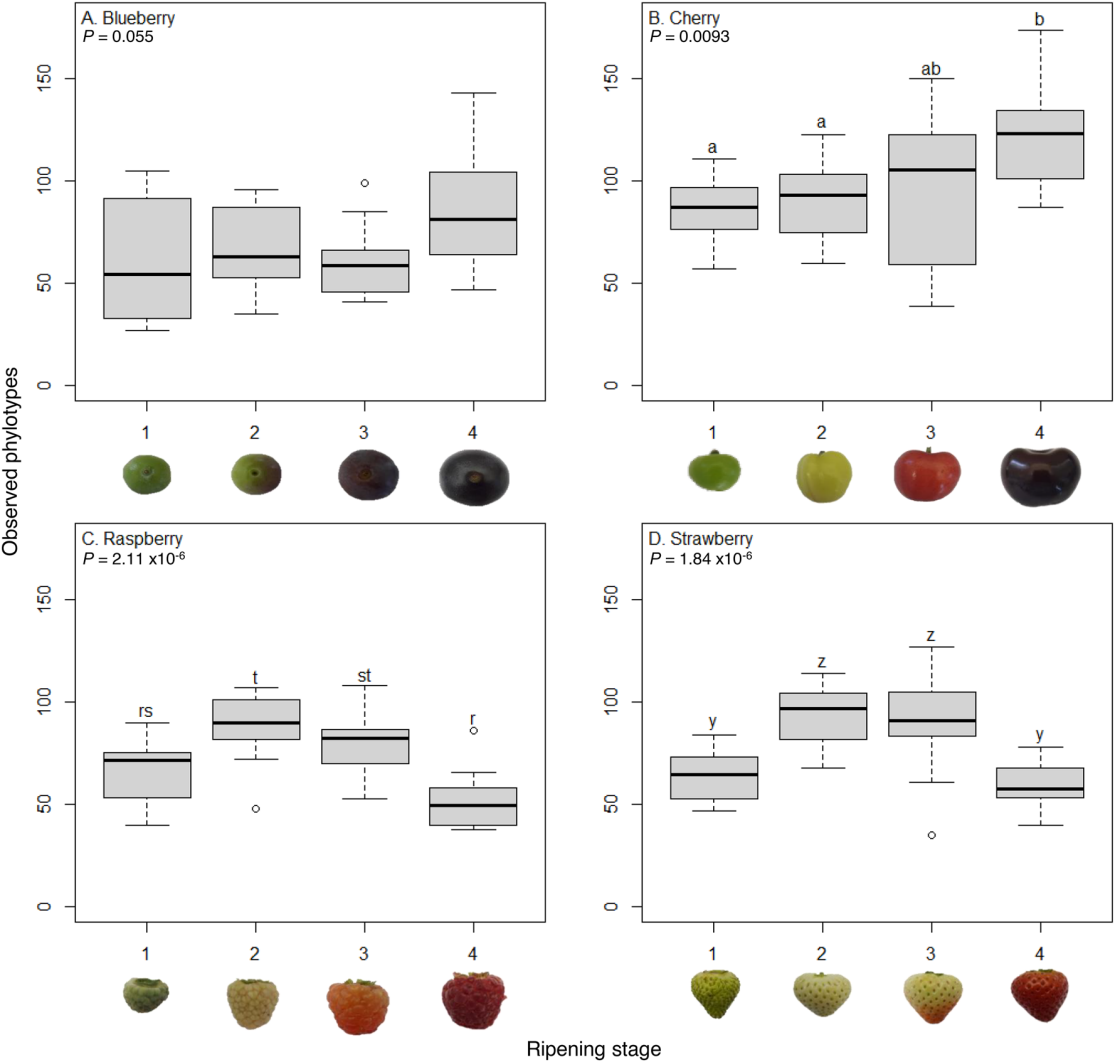
Number of observed phylotypes across fruit types and maturation stages. Number of fungal phylotypes across four ripening stages (1, unripe/green fruit; 2, de-greening fruit; 3, ripening fruit; and 4, fully ripe/harvest fruit) for blueberry, cherry, raspberry and strawberry (N = 12 except N = 11 for strawberry stage 3). Numbers of fungal phylotypes differ across ripening stages for cherry, raspberry and strawberry but not blueberry (ANOVA, *P* values shown). Where significant, different lowercase letters represent significant differences in phylotype numbers within each fruit (*P* < 0.028) with separate Dunn’s comparisons post-hoc (with Benjamini–Hochberg multiple comparison correction). Different letter groups show any significant differences between ripening stages within each fruit separately.

As there was a significant interaction between fruit and ripeness stage, we investigated the effect of ripening stage on each fruit separately. All data residuals were normally distributed (Shapiro–Wilks, *P* > 0.05) and there was a significant effect of ripening stage on the number of fungal phylotypes for cherry, raspberry, and strawberry (one-way ANOVA: *F*_3,44_ = 4.33, *P* = 0.0093; *F*_3,44_ = 13.56, *P* = 2.11 × 10^–6^ and *F*_3,44_ = 13.86, *P* = 1.84 × 10^–6^, respectively, Fig. 2), but not blueberry (*F*_3,44_ = 2.27, *P* = 0.055). On cherries phylotype numbers increased during ripening, but raspberry and strawberry had greater numbers at intermediate stages of fruit maturation (Fig. 2).

There was a significant effect of fruit type but not ripening stage on the number of Saccharomycetales budding yeast phylotypes (Kruskal–Wallis, chi-squared = 75.66, df = 3, *P* = 2.61 × 10^–16^ and chi-squared = 5.50, df = 3, *P* = 0.14 respectively). Raspberry (mean ± SE number of phylotypes = 12 ± 0.60) harboured significantly more Saccharomycetales phylotypes than strawberry (10 ± 0.74), cherry (7 ± 0.70), and blueberry (4 ± 0.31; Tukey’s HSD, *P* = 0.044, *P* = 2.9 × 10^–6^ and *P* = 1.5 × 10^–15^ respectively). Strawberry harboured significantly more phylotypes than cherry and blueberry (Tukey’s HSD, *P* = 0.007 and *P* = 2.6 × 10^–9^), and cherry harboured significantly more than blueberry (Tukey’s HSD, *P* = 0.001) (Fig. S5). Both Shannon’s and Simpson’s diversity indexes, which analyse the distribution of phylotype abundances, revealed differences between fruit species and ripening stage in line with the above findings (Table S2).

#### Presence/absence of fungal phylotypes

**Figure 3:**
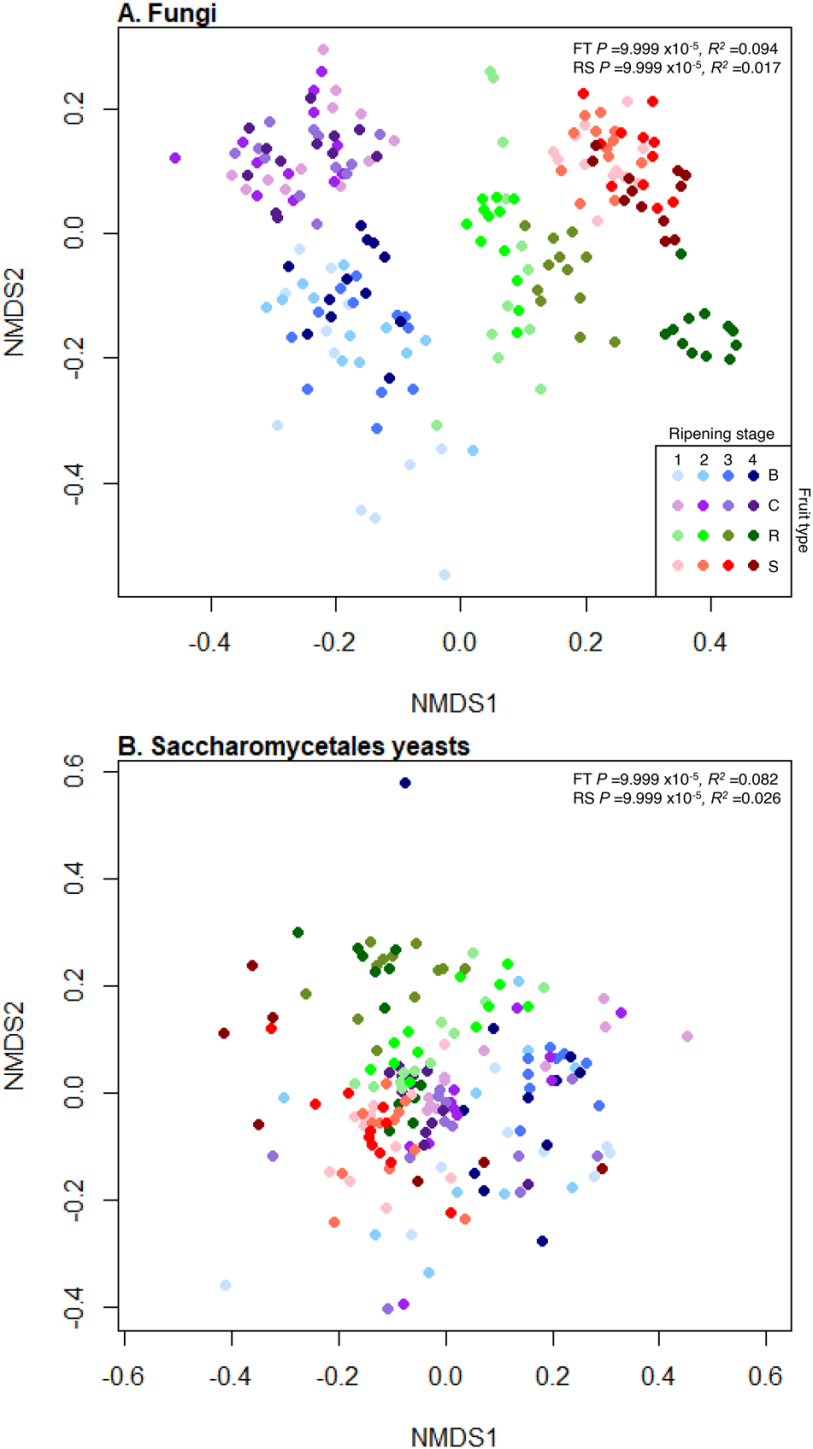
NMDS plots representing the differential presences of fungal phylotypes. Nonmetric Multidimensional Scaling (NMDS) plots of binary Jaccard measures of community dissimilarity of (**A**) total fungal communities and (**B**) Saccharomycetales budding yeasts on blueberry (blue), cherry (purple), raspberry (green) and strawberry (red) at four ripening stages (1, unripe/green fruit; 2, de-greening fruit; 3, ripening fruit; and 4, fully ripe/harvest fruit; denoted by shade of colour, lightest shade for green fruit and moving through to darkest shade for fully ripe/harvest). Both total fungal and Saccharomycetales yeasts communities significantly differ in the presences of phylotypes across all fruit types (FT) and ripening stages (RS) by PermANOVA (values shown top right).

Both fruit type and ripening stage significantly influenced the types of fungi present (PermANOVA, *R*^2^ = 0.094, *P* = 9.999 × 10^–5^ and *R*^2^ = 0.017, *P* = 9.999 × 10^–5^, respectively, Fig. 3A) and there was a significant interaction between fruit type and ripening stage (*R*^2^ = 0.013, *P* = 9.999 × 10^–5^). Comparisons of effect sizes (*R*^2^ values) showed fruit type had approximately 5.5 greater influence than ripening stage on the types of fungal phylotypes present. As there was a significant interaction between fruit and ripening stage, the effect of ripening stage on fungal communities was investigated for each fruit separately. Ripening stage significantly influenced the types of fungal phylotypes present on all fruit (blueberry *R*^2^ = 0.043, 9.999 × 10^–5^; cherry *R*^2^ = 0.060, *P* = 9.999 × 10^–5^; raspberry *R*^2^ = 0.13, *P* = 9.999 × 10^–5^ and strawberry *R*^2^ = 0.055, *P* = 9.999 × 10^–5^, Fig. S6). There were significant differences in presences of fungal phylotypes between all fruits and ripening stages (post-hoc pairwise PermANOVAs: *P* = 9.999 × 10^–5^, *R*^2^ range 0.09–0.20; Fig. 3A; Supplemental Tables S3, S4).

Both fruit type and ripening stage significantly influenced the types of Saccharomycetales phylotypes present (PermANOVA, *R*^2^ = 0.082, *P* = 9.999 × 10^–5^ and *R*^2^ = 0.026, *P* = 9.999 × 10^–5^, respectively, Fig. 3B) with a significant interaction between fruit type and ripening stage (*R*^2^ = 0.024, *P* = 9.999 × 10^–5^). In line with the general fungal community, comparisons of *R*^2^ values showed fruit type had approximately 3.15 times greater effect than ripening stage on the Saccharomycetales phylotypes present. Ripening stage significantly influenced the types of Saccharomycetales phylotypes on each fruit separately (blueberry *R*^2^ = 0.065, *P* = 0.0008; cherry *R*^2^ = 0.080, *P* = 0.0004; raspberry *R*^2^ = 0.27, *P* = 9.999 × 10^–5^ and strawberry *R*^2^ = 0.084, *P* = 9.999 × 10^–5^). There were significant differences in presences of different Saccharomycetales yeast phylotypes between all fruits and ripening stages (post-hoc pairwise PermANOVAs: *P* = 9.999 × 10^–5^, *R*^2^ range 0.06–0.15; Supplemental Tables S5, S6).

#### Relative abundances of fungal phylotypes

**Figure 4:**
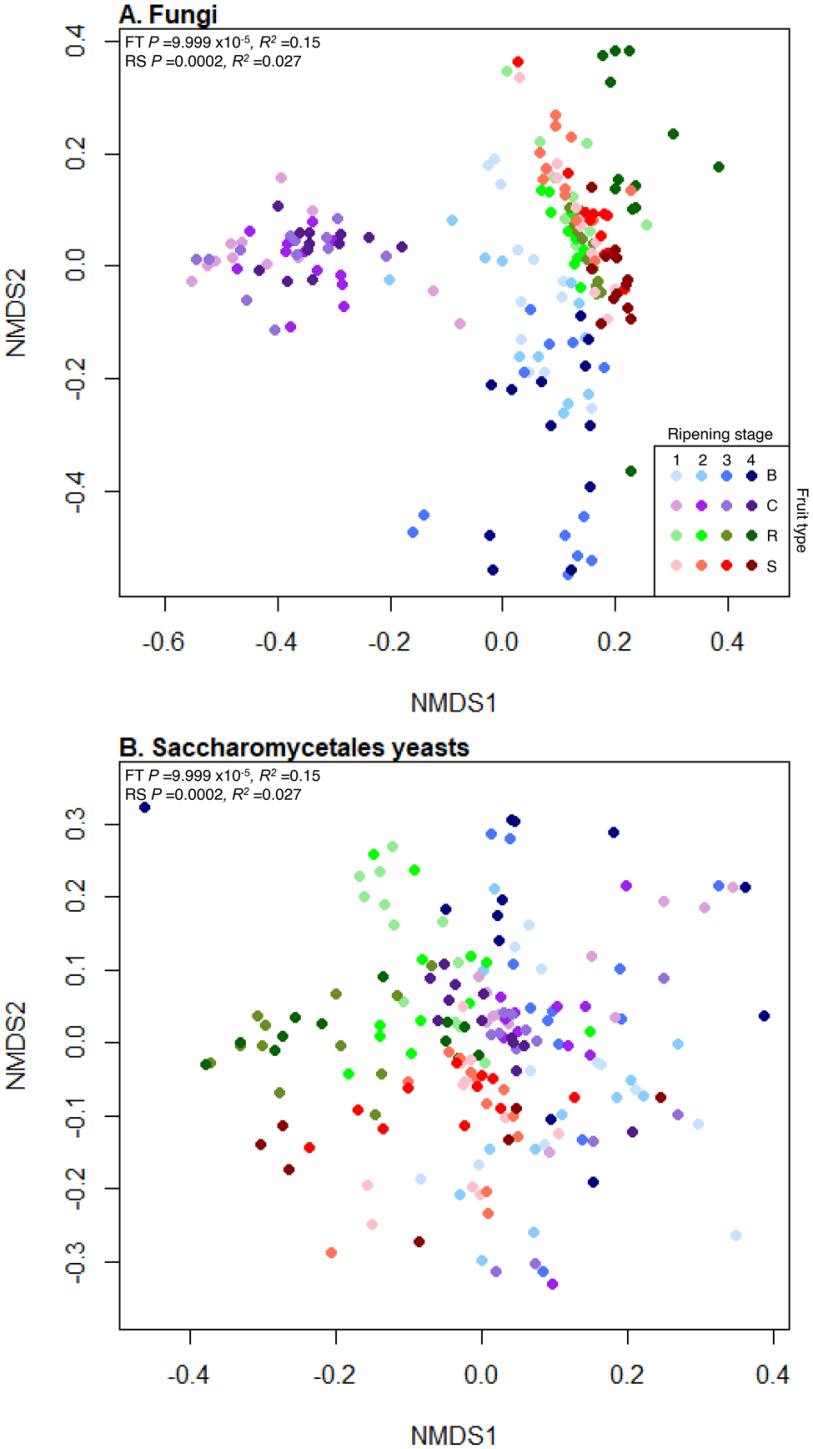
NMDS plots representing the differential abundances of fungal phylotypes. Nonmetric Multidimensional Scaling (NMDS) plots of abundance Jaccard measures of community dissimilarity of (**A**) total fungal communities and (**B**) Saccharomycetales budding yeasts on blueberry (blue), cherry (purple), raspberry (green) and strawberry (red) at four ripening stages (1, unripe/green fruit; 2, de-greening fruit; 3, ripening fruit; and 4, fully ripe/harvest fruit; denoted by shade of colour, lightest shade for green fruit and moving through to darkest shade for fully ripe/harvest). Both total fungal and Saccharomycetales yeasts communities significantly differ in the presences of phylotypes across all fruit types (FT) and ripening stages (RS) by PermANOVA (values shown top left).

Fruit type and ripening stage also significantly influenced the relative abundances of different fungal phylotypes (PermANOVA, *R*^2^ = 0.15, *P* = 9.999 × 10^–5^ and *R*^2^ = 0.027, *P* = 0.0002, respectively, Fig. 4A), and the interaction between them was also significant (*R*^2^ = 0.018, *P* = 0.003). Fruit type had approximately 5.6 times greater influence than ripening stage on the relative abundances of fungal phylotypes. Ripening stage significantly influenced the relative abundances of fungal phylotypes present on each fruit separately (blueberry *R*^2^ = 0.16, *P* = 9.999 × 10^–5^; cherry *R*^2^ = 0.061, *P* = 0.009; raspberry *R*^2^ = 0.24, *P* = 9.999 × 10^–5^ and strawberry *R*^2^ = 0.15, *P* = 9.999 × 10^–5^, Fig. S7). There were significant differences in fungal community composition between all fruits and ripening stages (post-hoc pairwise PermANOVAs: *P* = 9.999 × 10^–5^, *R*^2^ range 0.11–0.57; Supplemental Tables S7, S8).

Fruit type and ripening stage significantly influenced the relative abundances of Saccharomycetales phylotypes (PermANOVA, *R*^2^ = 0.038, *P* = 9.999 × 10^–5^ and *R*^2^ = 0.024, *P* = 9.999 × 10^–5^, respectively, Fig. 4B), with an interaction between the main effects (*R*^2^ = 0.016, *P* = 9.999 × 10^–5^). Fruit species had approximately 1.6 times greater influence than ripening stage on the relative abundances of phylotypes. Ripening stage significantly affected the relative abundances of Saccharomycetales phylotypes on each fruit separately (blueberry *R*^2^ = 0.043, *P* = 0.004; cherry *R*^2^ = 0.64, *P* = 0.003; raspberry *R*^2^ = 0.19, *P* = 9.999 × 10^–5^ and strawberry *R*^2^ = 0.070, *P* = 0.0009). There were significant differences in Saccharomycetales community composition between all fruit species and ripening stages (post-hoc pairwise PermANOVA: *P* = 9.999 × 10^–5^, *R*^2^ range 0.038–0.10; Supplemental Tables S9, S10).

### The similarities and differences of fungal phylotypes

#### The core fruit fungal microbiome

**Figure 5:**
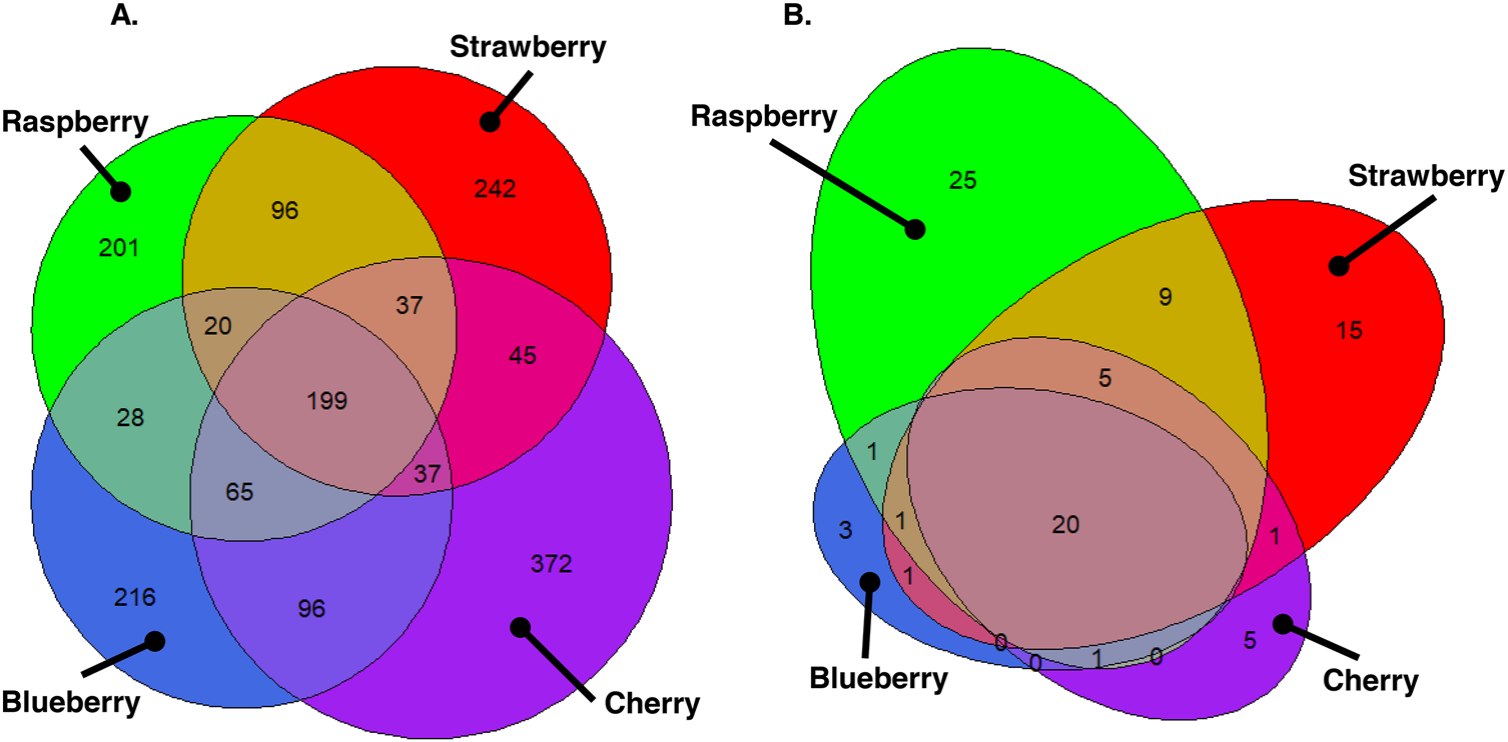
Comparison of shared and discrete numbers of fungal and Saccharomycetales phylotypes between fruits. Venn diagrams of phylotype counts across fruit types for (**A**) all fungal phylotypes, and (**B**) Saccharomycetales phylotypes; overlapping segments are approximately proportional to values.

Analyses thus far have focussed on differences in fruit microbiomes, but it is valuable to contrast this with quantifying fruit microbiome similarity. The core fruit fungal microbiome (i.e. those phylotypes present across all fruits) consisted of 199 (11.6%) of the 1712 fungal phylotypes and comprised 97.6% of DNA reads (Table S11). Approximately 12–22% of the 1712 phylotypes were only found associated with specific fruits: 216 with blueberry, 372 with cherry, 201 with raspberry, and 242 with strawberry (Fig. 5A, Table S11). Twenty of the 87 Saccharomycetales phylotypes (23.0%) comprising 81.2% of Saccharomycetales reads were present across all fruit types (Table S11), with 3 unique to blueberry, 5 to cherry, 25 to raspberry and 15 to strawberry (Fig. 5B, Table S12).

#### The phylotypes that are most differentially abundant

Analyses across all biodiversity metrics show fruit type had a greater effect on fungal communities than maturation stage. Overall, 195 (11.4%) indicator phylotypes (spanning 76 families) had significantly differential abundances between fruit types: 33 phylotypes were significantly overrepresented on blueberry, 70 on cherry, 39 on raspberry and 53 on strawberry (FDR corrected *P* values ranging from *P* = 0.011 to *P* = 0.044). The complete list of significantly differentially overrepresented phylotypes is shown in Table S13 but the two most significantly differentially overrepresented phylotypes on each fruit are listed here: *Polyphialoseptoria* species and *Ramularia* (most likely *Ramularia endophylla*) on blueberry; *Exobasidium* species and a phylotype from the poorly described order Leotiomycetes on cherry; phylotypes with > 97% homology to *Metschnikowia kunwiensis* and *H. uvarum* on raspberry; and phylotypes with > 97% homology to *Kalmanozyma fusiformata* (*Ustilaginaceae* smut fungi) and *Podosphaera aphanis* on strawberry.

**Figure 6:**
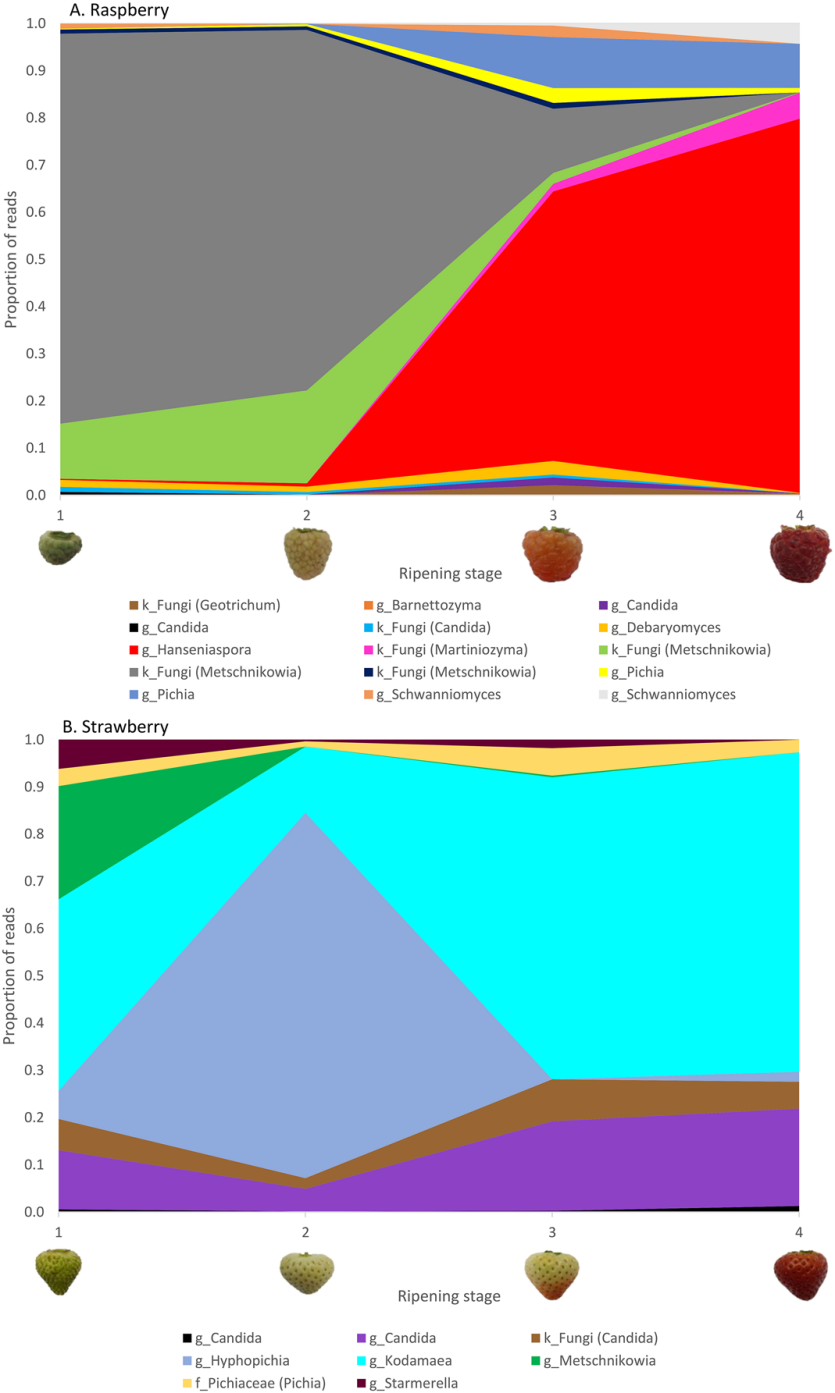
Dynamics of changes in the proportion of budding yeast indicator phylotypes. Mean proportion of reads for the Saccharomycetales budding yeast indicator phylotypes that are significantly overrepresented on (**A**) raspberry and (**B**) strawberry (*P* < 0.04) across the four ripening stages (1, unripe/green fruit; 2, de-greening fruit; 3, ripening fruit; and 4, fully ripe/harvest fruit). Indicator phylotypes are reported to the taxonomic level assigned: lower case letter refers to the taxonomic hierarchy of respective taxa (g = genus; f = family; k = kingdom). Where possible, assignment to genus taxonomic levels is shown in parentheses from matches to deposits in Genbank with > 97% homology identified by manual Blast searches.

Twenty-four of the 195 indicator phylotypes belonged to the Saccharomycetales budding yeasts (Table S13). There were no Saccharomycetales indicator phylotypes for cherry, and just one for blueberry, a fungal phylotype with > 97% homology to *Metschnikowia koreensis.* Raspberry had 15 Saccharomycetales indicator phylotypes: three with > 97% homology to the *Metschnikowia* and, *Candida* genera, two *Pichia* and *Schwanniomyces,* and one each from *Hanseniaspora, Barnettozyma*, *Debaryomyces, Candida, Geotrichum* and *Martiniozyma*. There were eight indicator phylotypes for strawberry; two *Candida* and one from each of the *Metschnikowia*, *Starmerella*, *Kodamaea* and *Hyphopichia* genera and the *Pichiaceae* family, and a phylotype assigned to the no higher level than fungal kingdom (with > 97% homology to deposit from *Candida* genus). The dynamics of Saccharomycetales yeast indicator phylotypes abundances across maturation for raspberry and strawberry is shown in Fig. 6.

#### Differences of yeast known to be attractive to *D. suzukii*

Yeast from the *Hanseniaspora, Pichia, Saccharomyces*, *Candida* and *Metschnikowia* genera and their combinations are attractive to *D. suzukii*^27,28,30,31^, and phylotypes belonging to these genera were recovered here. The combined relative read abundances of all phylotypes assigned to these genera were significantly different between fruit types and ripening stages (Kruskal–Wallis chi-squared = 60.54, *P* = 4.51 × 10^–13^; chi-squared = 10.11, *P* = 0.018, respectively). Raspberry had the highest relative abundance of yeast genera known to be attractive to *D. suzukii* (mean ± SE = 21,539 ± 4339) and this was significantly greater than on the other fruits (*P* < 1.97 × 10^–8^): cherry (1535 ± 265), strawberry (1651 ± 234) and blueberry (8009 ± 2648). When fruit types were analysed individually, ripening stage had a significant effect on relative read abundance of attractive yeast genera for raspberry only (Kruskal–Wallis chi-squared = 28.70, *P* = 2.59 × 10^–6^) where stage 1 and 4 abundances did not significantly differ (mean ± SE = 5682 ± 1522 and 20,826 ± 4711 respectively) but were significantly greater than stage 2 and 3 (2163 ± 538 and 4113 ± 1494 respectively; *P* < 0.05; Fig. S8).

Various isolates of *H. uvarum* have consistently been shown to be attractive to *D. suzukii*^27,28,30–32^. Seven phylotypes were assigned to *Hanseniaspora* and four of these had > 97% homology to *H. uvarum* deposits in Genbank using Blast searches, and the relative abundance of these four phylotypes significantly differed between fruit types (Kruskal–Wallis chi-squared = 70.67, df = 3, *P* = 3.08 × 10^–15^). Raspberry had the highest relative abundance of *H. uvarum* (mean ± SE 13,843 ± 3991) but this was not significantly greater (*P* = 0.080) than abundances on strawberry (426 ± 134) but was significantly greater than blueberry (6 ± 4) and cherry (8 ± 2; *P* = 1.62 × 10^–12^, *P* = 6.00 × 10^–9^ respectively). Raspberry and strawberry were the only fruits where maturation stage had a significant effect on *H. uvarum* relative abundance (Kruskal–Wallis chi-squared = 33.40, df = 3, *P* = 2.66 × 10^–7^; chi-squared = 12.59, df = 3, *P* = 0.006), and *H. uvarum* relative abundance increased as fruits ripened (Fig. S9). This analysis is in line with the indicator phylotype analysis which reported a *Hanseniaspora* phylotype with > 97% homology to *H. uvarum* as over-represented on raspberry generally, and especially at later stages (Fig. 6A).

#### Differences of *Botrytis cinerea*, known to be repulsive to *D. suzukii*

The relative read abundances of *B. cinerea* were significantly different between fruit types and ripening stages (Kruskal–Wallis chi-squared = 73.45, *P* = 7.80 × 10^–16^; Kruskal–Wallis chi-squared = 23.81, *P* = 2.74 × 10^–5^, respectively). Raspberry had the lowest relative abundance of *B. cinerea* (mean ± SE = 800 ± 136) and this was significantly lower than strawberry (1994 ± 292) and blueberry (5990 ± 1305) (*P* < 0.004), but not cherry (3015 ± 1406). Cherry and strawberry had significantly lower reads than blueberry (*P* < 4.13 × 10^–5^). When fruit types were analysed individually, ripening stage had a significant effect on relative read abundance of *B. cinerea* for all fruits (*P* < 0.003) and reads generally increased during ripening except on cherry where stage 2 had the greatest (Fig. S10).

#### Correlations with fruit host potential index (HPI) scores

Finally, Bellamy et al.^38^ generated fruit host potential index (HPI) scores from interactions of *D. suzukii* with commercial ripe fruit including the fruit species analysed here. The combined relative abundances (i.e. the total number of reads on each fruit across replicates) of yeast phylotypes empirically shown to be attractive to *D. suzukii* (*Hanseniaspora, Pichia, Saccharomyces*, *Candida* and *Metschnikowia*^27–31^) across these different ripe fruits at the last sample point are positively correlated with fruit HPI scores (Pearson’s correlation *r* = 0.38), as are the relative abundance of just *H. uvarum* (*r* = 0.62). The relative abundance of *B. cinerea* was negatively correlated to HPI scores (*r* =  − 0.65) (Fig. S11); however, none of these correlations were significant (*P* > 0.35) likely due to the low number of comparisons (N = 4 due to just one HPI score per fruit type).

## Discussion

*Drosophila suzukii* is attracted to fungal volatile chemicals (e.g.^27^); however, little is known about the fungal microbiomes of commercial fruit, with a paucity of information for *D. suzukii* susceptible fruit. Here we tested the hypothesis that both fruit type and maturation stage have a significant effect on total fruit fungal communities as well as Saccharomycetales yeasts and found strong support for this for all three community biodiversity metrics analysed (numbers, types, and abundances of phylotypes). Raspberry had the greatest relative abundance of yeasts known to be attractive to *D. suzukii.* Overall, there was a fivefold greater difference in fungal communities between fruit types than maturation stages, showing fruit type was the greater factor defining fruit fungal community assemblage, and cherry had the most distinctive fungal microbiome (Fig. 5). However, there are two main caveats to these conclusions which need consideration. First, we note that fruits matured across different absolute time periods meaning the absolute timing of sampling the various maturation stages differed between fruits, and there is some evidence that other fruit microbiomes can differ through time^39,40^. Second, fruit fungal communities have been shown to differ by geographic location across hundreds of kilometres^6,10,12^ and at smaller scales: for example, fungal community dissimilarity increased with distance on grapes from six vineyards separated by a maximum of 35 km^11^. Therefore, as different fruit sampled in this study were from separate locations up to 19 km apart, it is possible that the variance in fruit-associated fungal microbiomes was also influenced by geographic location. There is support for greater microbiome differentiation by distance from a simple correlation of geographic and community differentiation distances (*P* = 0.001; Mantle test on distance in km and Jaccard distance); however, distance does not completely explain the variation in fruit microbiomes as cherry and raspberry fungal communities have the greatest dissimilarity (are most separated on NDMS 1 in Fig. 4) but derive from some of the most closely geographically situated sites (Fig. S12). Different fruits would have to be sampled from immediately adjacent sites to completely discount any effect of geographic location on fungal microbiomes. Additionally, while all sites were under conventional management approaches, the precise details of spray programmes are commercially confidential, so it is possible that sites with different fruits were treated with differing spray programmes to control for pest and diseases including fungicides, which may have influenced fungal microbiomes. Some evidence from wine grapes in New Zealand suggests the differences between conventional and biodynamic management only has a small effect on fruit fungal microbiomes^14^. However, other studies on grapes and pear flowers reported that management practices had a significant effect on total culturable yeasts as well as on community structure^13,41^. Taken together, the effect of fruit-type detected in this study is likely to be a composite effect of complex interactions of fruit-type × location × management practices and further study on the same fruit species across multiple locations would be necessary to confirm the extent to which fruit species impact microbial communities. Another caveat is that the inference of fungal biodiversity here is derived from the analysis of DNA, and this may not necessarily correlate with phylotypes which are active in communities, and the complementary analyses of RNA may provide an insight into this.

Regardless of the above caveats, these findings show differences in fungal communities on commercial fruit in space and time, and this also holds for species implicated in the attraction of the *D. suzukii* insect pest. These findings are in line with the few other studies in this field which have shown that fungi differ significantly between apples and blackcurrants^4^, as well as between sea buckthorn, black chokeberry, red and white currants^3^. While differences in fungal communities across ripening stages were smaller here, they still changed significantly, especially in the types and abundances of phylotypes, and this agrees with the very limited data from a few other studies evaluating the dynamics of microbiomes as fruit matures^8,9,36^. However, the temporal dynamics differed between fruit types: numbers remained constant for blueberry but increased with ripening for cherry, and the intermediate ripening stages of raspberry and strawberry had more phylotypes (Fig. 2). Ripening fruit represents a changing habitat which undergoes several physiological changes, including an increase in size and sugar content as well as changes in firmness, colour, and other secondary metabolites which may contribute to fungal community composition. Despite revealing differences in fungal communities between fruit types/sites and maturation stages, there was a large core microbiome which was present across all fruits/sites: this comprised only a fraction of diversity at just 199 of the 1712 fungal phylotypes (Table S11) but was the majority in terms of abundance as it comprised 97.6% of the DNA reads.

Our attempt to quantify changes in absolute fungal cell numbers was only successful for cherry. The total fungal population load per mm^2^ remained constant across ripening, but there are no other published quantitative DNA based estimates from fruit for comparison. Further optimisation of the levels of added internal standard cells may have allowed quantitative estimates across all fruits; alternatively, adding a synthetic chimeric DNA spike to samples before DNA extraction may be a better strategy^42^. Using synthetic sequences as an internal standard has the added benefit of this not occurring in environmental samples^35^. It is also worth noting that including an internal standard in the form of live cells added before DNA extraction assumes that DNA extraction and amplification will be the same across all fungal cells present.

The nature of differences observed for total fungal communities generally held when just Saccharomycetales yeasts were analysed. Specific Saccharomycetales yeast genera which have been empirically shown to be attractive to *D. suzukii* in field and lab assays^27,30^ were more prevalent at the raspberry site. Further, the species which has been implicated most in *D. suzukii* attraction, *H. uvarum*^32^, was highly abundant on raspberry. In addition, *B. cinerea* has been shown to have a repellent effect for *D. suzukii*^34^. Of the four fruit sites, raspberry had the lowest amounts of *B. cinerea* showing an inverse correlation with yeasts attractive to *D. suzukii*. Raspberry was also the fruit with the greatest host potential index scores for *D. suzukii* attraction by Bellamy et al.^38^, and together these observations are in line with the hypothesis that *H. uvarum* plays a role in *D. suzukii* attraction to fruit. However, it must be noted that the observed correlation between yeasts shown in other work to be attractive to *D. suzukii* and the abundances of these yeasts on fruit shown in this study cannot be taken as a causative correlation at this stage. There are other factors like fruit acidity, sugar content and firmness that have been shown to influence in fruit preference of *D. suzukii*^43–45^. Further work is needed to directly empirically determine the extent to which yeast communities affect *D. suzukii* preference for fruit. As it stands, these are general correlations, and one may not yet conclude that these abundant yeast phylotypes necessarily drive attraction. The observations here may be compared with a study showing that greater numbers of *D. suzukii* larvae developed on strawberries than raspberries which where greater than on blueberry; however, this study did not control for fruit associated microbiomes, and factors other than yeast communities may have caused these differing observations^46^.

Overall, further work is needed to understand if such fruit microbial patterns hold in other locations at other times and whether this correlation with attractive yeast from laboratory and field assays has any underlying basis for causation for *D. suzukii* fruit susceptibility in the field. If so, this opens the possibility of manipulating fruit microbiomes to deter *D. suzukii.* Whether fungal species repulsive to *D. suzukii* species could be ‘seeded’ onto fruits to reduce attractiveness is an intriguing question. Similarly, if this could be combined with traps containing attractive baits situated in and around crops to form a push–pull system to push flies away from fruits and attract them into traps. Although it is unrealistic to use *B. cinerea* in this way due to its phytopathogenic nature, certain yeast species are known to be repulsive to *D. simulans* and *D. melanogaster*^30,47–49^. Van Timmeren et al.^50^ demonstrated that crop sterilants also impact attractive yeast species growth and reduce *D. suzukii* larval infestation of fruit. A logical extension of this implies that future data might reveal specific microbes, which are not harmful to fruits or humans and are able to reduce *D. suzukii* attraction and could therefore be applied for crop protection.

## Conclusion

This study demonstrates that for general fungal and more specific Saccharomycetales yeast communities, fruit type or site and maturation stage have a significant impact on fungal diversity, with fruit type/site having a larger effect. This observation also holds for yeast species known to attract *D. suzukii,* and here these yeasts were most abundant on raspberry^30^. This knowledge may potentially be applied to better understand what drives *D. suzukii* susceptibility of different fruit crops at different sites. It is also possible this may inform the engineering of fungal/ yeast communities which could be ‘seeded’ on fruit to reduce the susceptibility of commercial fruit crops to *D. suzukii*, or to identify ecologically realistic yeast communities for use as potentially attractive phagostimulant baits to control to *D. suzukii* and reduce the use of chemical pesticides.

## Materials and methods

### Fruit sampling and processing

All methods, including fruit collections, were performed in accordance with relevant guidelines and the project was conducted under ethics approval CoSREC388 from the University of Lincoln. Based on fruit pigmentation, blueberries, cherries, raspberries, and strawberries were sampled at four developmental stages ranging from unripe (green) to fully ripe (red/purple/navy) (Table S14, Fig. S1) throughout June to September in 2018. Sampling times differed for each fruit type (Table S14). All samples were collected from commercial fruit growers in the United Kingdom southern county of Kent at a maximum distance of 19 km apart; the same sites were revisited at each ripening stage. All fruit were subject to growers’ spray programmes to control pest and diseases. Ten fruits (except blueberries N = 20 as these are smaller) were collected for each species and combined into one sterile bag, and this was replicated six times within each site at each of the four stages for each fruit, totalling 1200 individual and 96 combined fruit samples. Fruits were randomly selected within each field or orchard and were aseptically removed with as little of the stalk or calyx as possible without damaging the fruit. Fruits were briefly inspected for damage before removal with sterile scissors, and fruits were allowed to drop directly into sterile sample bags and thus not handled. Fruits were transported directly to the laboratory where 20 mL of sterile water was added to sample bags. Fruits were then surface-washed repeatedly with this water for 15 s every 5 min for 30 min, after which the contents collected in sterile 50 mL falcon tubes and centrifuged for 30 min at 4500 rpm to collect microbes. No surfactants were used in the surface washing process, as fungi vary in their hydrophobicity this may have affected isolation of certain fungi. The supernatant was reduced to approximately 2 mL, the pellet re-suspended and 1 mL was transferred to microfuge tubes and centrifuged further at 13,000 rpm for 10 min. The supernatant was discarded, and the pellet stored at − 80 °C. After washing, fruit were measured with vernier callipers and surface area estimated using 4πr^2^.

### DNA extraction

Pellets derived from samples were thawed and re-suspended in sterile water, then split into two equal parts. One half of each sample was spiked with 265 live *P. cucumerina* (Ascomycete: Sordariomycetes) cells determined using a haemocytometer to act as an internal standard. This constituted pairs of samples which were identical other than the spiked *P. cucumerina* internal standard cells to allow an estimate of absolute cell numbers in the resulting sequence data. *Plectosphaerella cucumerina* has rarely been reported on the surface of fruits and the isolate used derived from pumpkins in Lincolnshire (UK) and was grown in potato dextrose broth (ThermoFisher Scientific) at 25 °C for 7 days prior to use. Direct cell counts from these fruit samples indicated that 265 cells would represent approximately 0.5% of the community and thus be detectable. DNA was extracted using the DNeasy Blood and Tissue kit (QIAGEN) following the manufacturer’s instructions but with an additional bead beating step before incubation: pellets were resuspended in 750 µL ATL lysis buffer and added to 1 g of sterile glass beads with a 1:1 ratio of < 106 µm: 0.5 mm size (Sigma-Aldrich), then placed in a bead beater (Bead Ruptor 12, Omni international INC) at maximum speed for 5 × 30 s.

### Barcode amplification

PCR reactions comprised 15 µL Kapa 2× master mix (Kapa Biosystems), 6 µL of ITS2 forward and reverse primers with Illumina adaptors TS3_KYO2^51^ and ITS4^52^ modified with MiSeq adapters, 7 µL sterile water and 2 µL template DNA. Each batch of PCR reactions included a negative (2 µL sterile water) and positive (*S. cerevisiae* DNA) control. The PCR cycle parameters were 95 °C for 3 min, 29 cycles of 98 °C for 20 s 64 °C for 20 s 72 °C for 40 s, followed by a final extension time at 72 °C for 5 min. PCR products were separated by electrophoresis using 2% agarose gels containing 10 µL SYBR safe dye™ (Invitrogen) per 100 mL TAE (Tris Acetate-EDTA) buffer (ThermoFisher Scientific). PCR amplicons were sequenced on Illumina MiSeq instruments with a 300PE metric by Eurofins genomics. Raw sequences are deposited on SRA with the following project ID: PRJNA732273.

### Bioinformatics analysis

DNA sequences were processed with QIIME 2 (2019.4)^53^. Sequence quality was evaluated with FastQC^54^ and reads were trimmed, denoised, paired end merged and ASVs identified with DADA2^55^. ASVs were subsequently clustered with a > 97% genetic identity using vsearch^56^, and we term ASVs with > 97% identity ‘phylotypes’. Phylotypes assigned to the fungal kingdom were identified using q2-feature-classifier plugin using the unite_ver7dynamic database^57^; any unassigned phylotypes were subjected to manual Blast searches against the Genbank nucleotide database, and only phylotypes identified as belonging to the fungal kingdom were retained. For non-quantitative analysis, any phylotypes with 100% identity to the *P. cucumerina* internal standard were removed. Raw sequence counts were subjected to CSS variance‐stabilising normalisation using metagenomSeq and phyloseq R packages^58–61^. Recent work indicates that analyses with equal sample depths by rarefication produces the same general patterns as with CSS variance‐stabilising normalisation^62^, and this is especially important for comparisons of species richness among samples. For the quantitative analysis of fungal communities, samples containing the spiked fungal internal standard (*P. cucumerina*) were separately processed through the bioinformatics pipeline. Quantitative estimates of phylotype cell counts were calculated by normalising the read number of each phylotype to the number of *P. cucumerina* reads in that sample, and absolute cell numbers estimated from the knowledge that 265 *P. cucumerina* cells were added. Phylotype assignments at the species level were estimated by Blast searching the Genbank nucleotide database with representative sequences and reporting hits with > 97% homology. The order Saccharomycetales was analysed by filtering for all phylotypes assigned to Saccharomycetales at the order level.

### Statistical analysis

R version 3.6.1 was used for all statistical analyses^63^. The effect of fruit species and ripening stage on numbers of phylotypes (richness) was assessed using a two-way ANOVA with Tukey HSD for post-hoc pairwise comparisons. A square root transformation was applied where the data did not conform to the assumption of normality as determined by Shapiro-Wilks tests, and Kruskal–Wallis tests applied if transformation did not achieve normality. Omega squared estimates of effect size for two-way ANOVA were calculated with ω^2^ = *df*_effect_ × (*MS*_effect_ − *MS*_error_)/(*SS*_total_ + *MS*_error_)^64^. Shannon’s and Simpson’s diversity indexes were analysed using Kruskal–Wallis tests. Differences in presences or absences of fungal phylotypes and relative abundances of phylotypes were analysed with two‐way full factorial permutational multivariate ANOVA (PermANOVA) using the ‘adonis’ function in the vegan package^65^ with 10,000 permutations on binary (phylotype presences) and abundance based Jaccard dissimilarity matrices^66^. Pairwise PermANOVAs were conducted to analyse differences within fruit species and ripening stages where required. For quantitative analysis of fungal communities, the effect of ripening stage on cell numbers was analysed using a Kruskal–Wallis test. Indicator analysis was used to determine fungal phylotypes which were over-represented in the different fruit species with the ‘indicspecies’ package^67^. ASV abundances were correlated to overall Host Potential Index scores taken from Ref.^38^ using Pearson’s correlation coefficient. Venn diagrams were created with the ‘eulerr’ package^68^. Mantel test was used to correlate geographic and community difference using vegan^65^.

### Ethics approval

This project was approved by the University of Lincoln ethics board (CoSREC388).

## Supplementary Information

Supplementary Information 1.

Supplementary Information 2.

## Data Availability

Raw sequences are available on SRA (project ID: PRJNA732273) and the ASV table is provided in the Supplementary Material.
