# Supplementary material for: The relative abundances of yeasts attractive to Drosophila suzukii differ between fruit types and are greatest on raspberries
Source: Sci Rep. 2022 Jun 20;12:10382. doi: 10.1038/s41598-022-14275-x (PMC9209449; doi:10.1038/s41598-022-14275-x)
Supplement: Supplementary file 1 — Supplementary Information 1. [file 41598_2022_14275_MOESM1_ESM.pdf]

Supplementary material: The relative abundances of yeasts attractive to *Drosophila suzukii* differ between fruit types and are greatest on raspberries.

Rory Jones[1,2]*, Michelle T. Fountain[2], Nadia A. Andreani[1,3], Catrin S. Günther[1,4] and Matthew R. Goddard[1,5]

[1]School of Life Sciences, University of Lincoln, Lincoln, LN6 7DL, UK.

[2]NIAB EMR, New Road, East Malling, Kent ME19 6BJ, UK.

[3]Present address: Max Planck for Evolutionary Biology, Plön, Germany.

[4]Present address: The New Zealand Institute of Plant and Food Research Ltd, Ruakura Research Campus, Bisley Road, Hamilton 3214, New Zealand.

[5]The School of Biological Science, University of Auckland, New Zealand.

*Corresponding author: r.jones26@live.co.uk

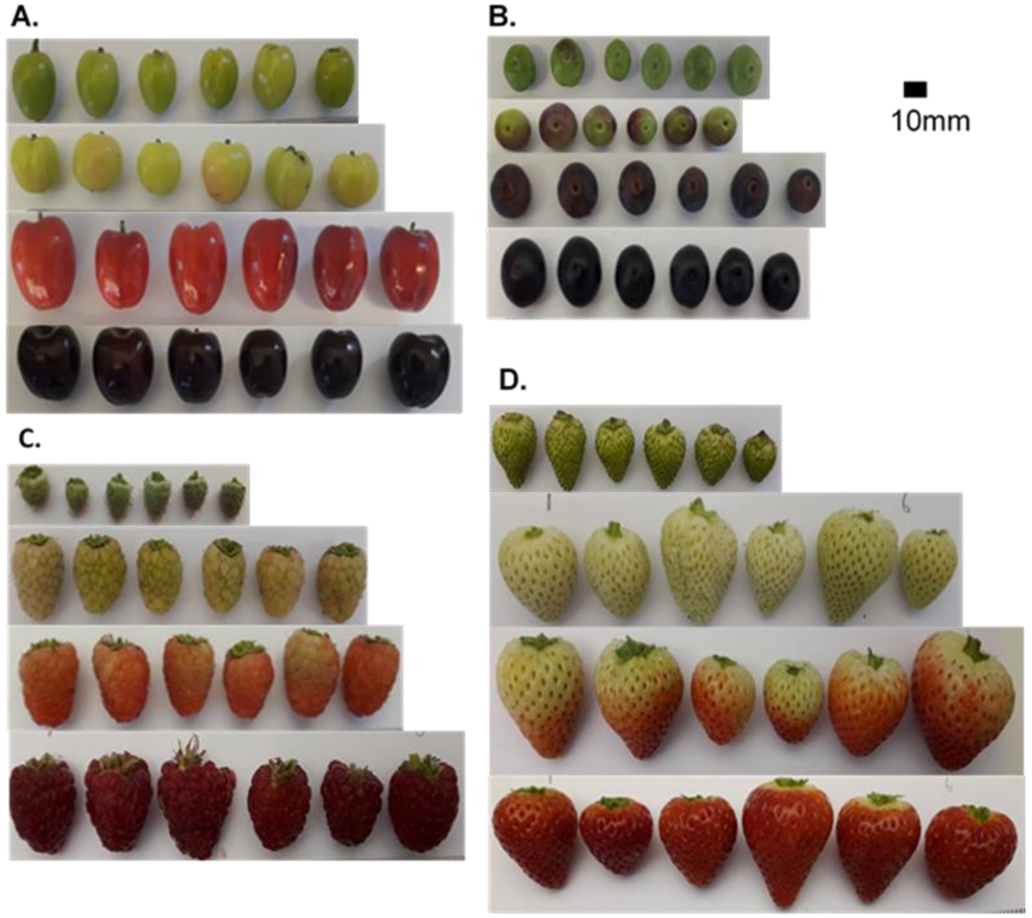

Fig. S1 The four ripening stages of each fruit sampled. A. green, white/pink, red and purple/harvest (top to bottom) of cherry. B. green, green/purple, purple and navy/harvest (top to bottom) of blueberry. C. green, white, pink and red/harvest (top to bottom) of raspberry, D. green, white and red/white (pink) and red/harvest (top to bottom) of strawberry.

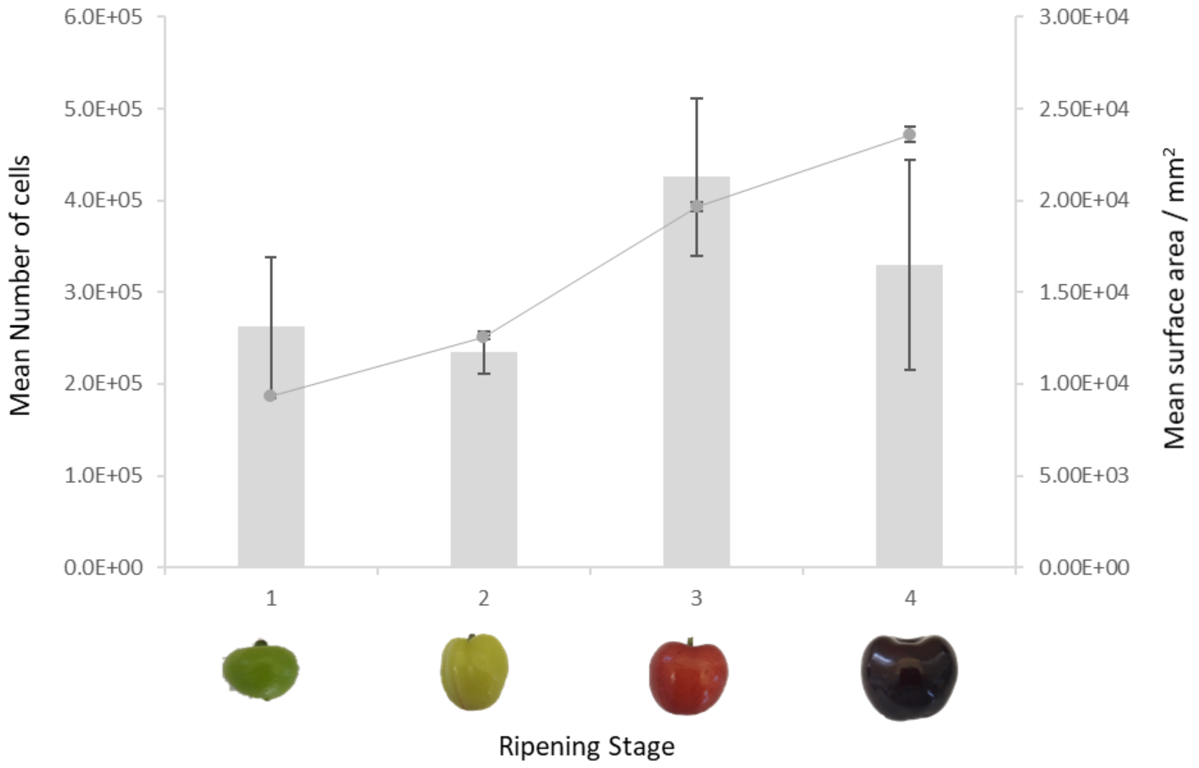

Fig. S2 Mean (±SE) number of fungal cells (left axis, bar plot) for cherry, not adjusted for surface area (N=6 except, stage 3 and 4, N=5). There was no significant effect of ripening stage on the number of fungal cells (Kruskal-Wallis, chi-squared = 2.63, P = 0.45), but there was a significant effect of ripening stage on cherry surface area (right axis, line chart) (Kruskal-Wallis, chi-squared = 19.70, P = 0.0002).

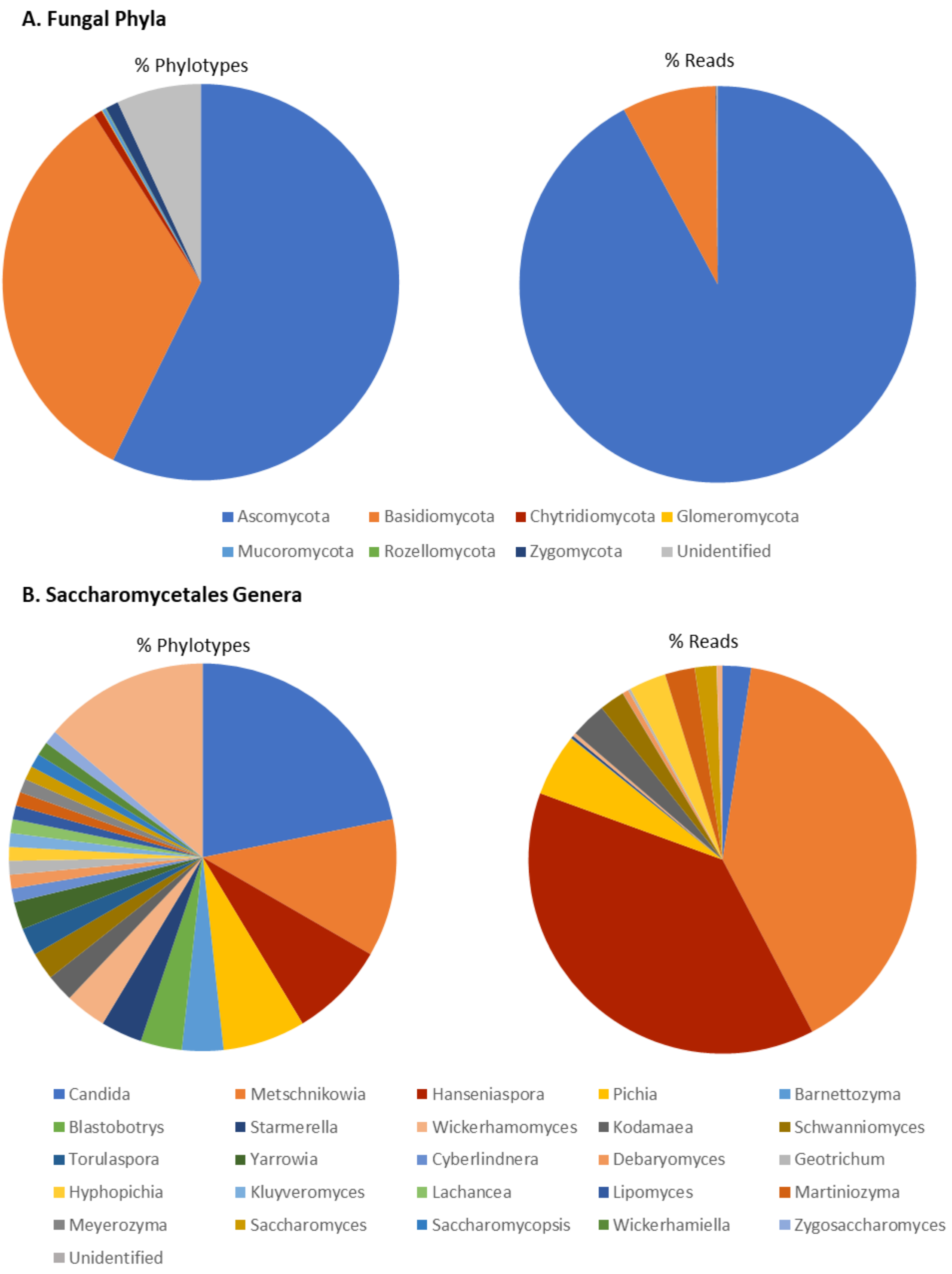

Fig. S3 (A.) Pie charts showing the proportion of both number of phylotypes (left) and reads (right) for the different fungal Phyla detected across all samples and (B.) pie charts showing the proportion of both number of phylotypes (Left) and reads (right) for the different Saccharomycetales yeast genera detected.

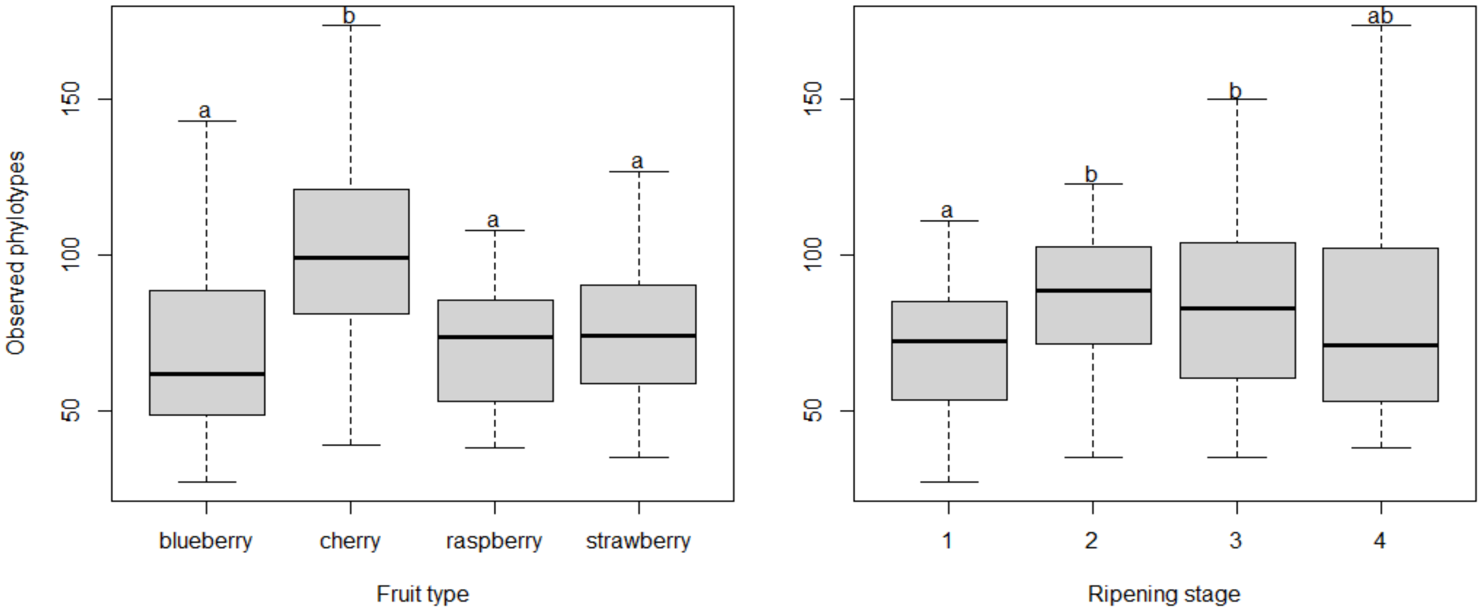

Fig. S4 Left, Number of observed fungal phylotypes present across the four fruit species. Right, average number of fungal phylotypes present across the four ripening stages (N=12 except strawberry stage 3 N=11). Different lowercase letters represent any significant difference in phylotype numbers between fruit type or ripening stages.

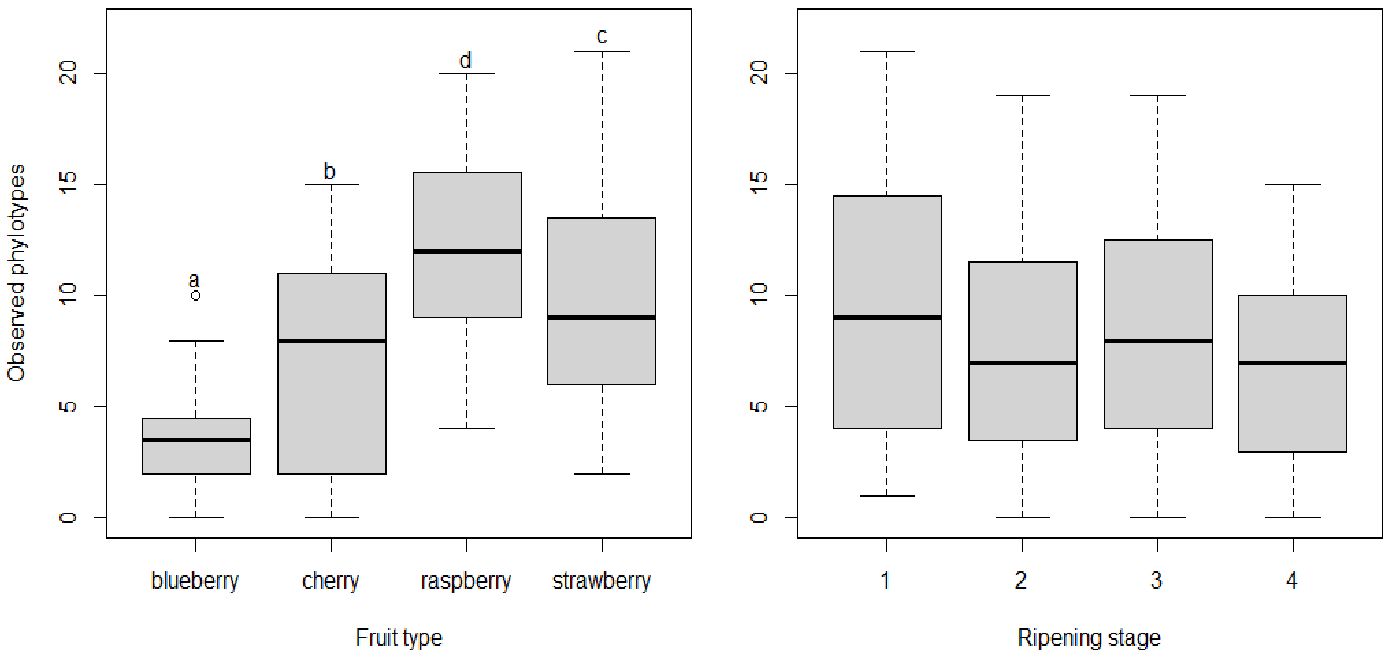

Fig. S5 Left, Number of observed Saccharomycetales budding yeast phylotypes present across the four fruit species. Right, average number of Saccharomycetales phylotypes present across the four ripening stages (N=12 except strawberry stage 3 N=11). Different lowercase letters represent any significant difference in phylotype numbers between fruit type.

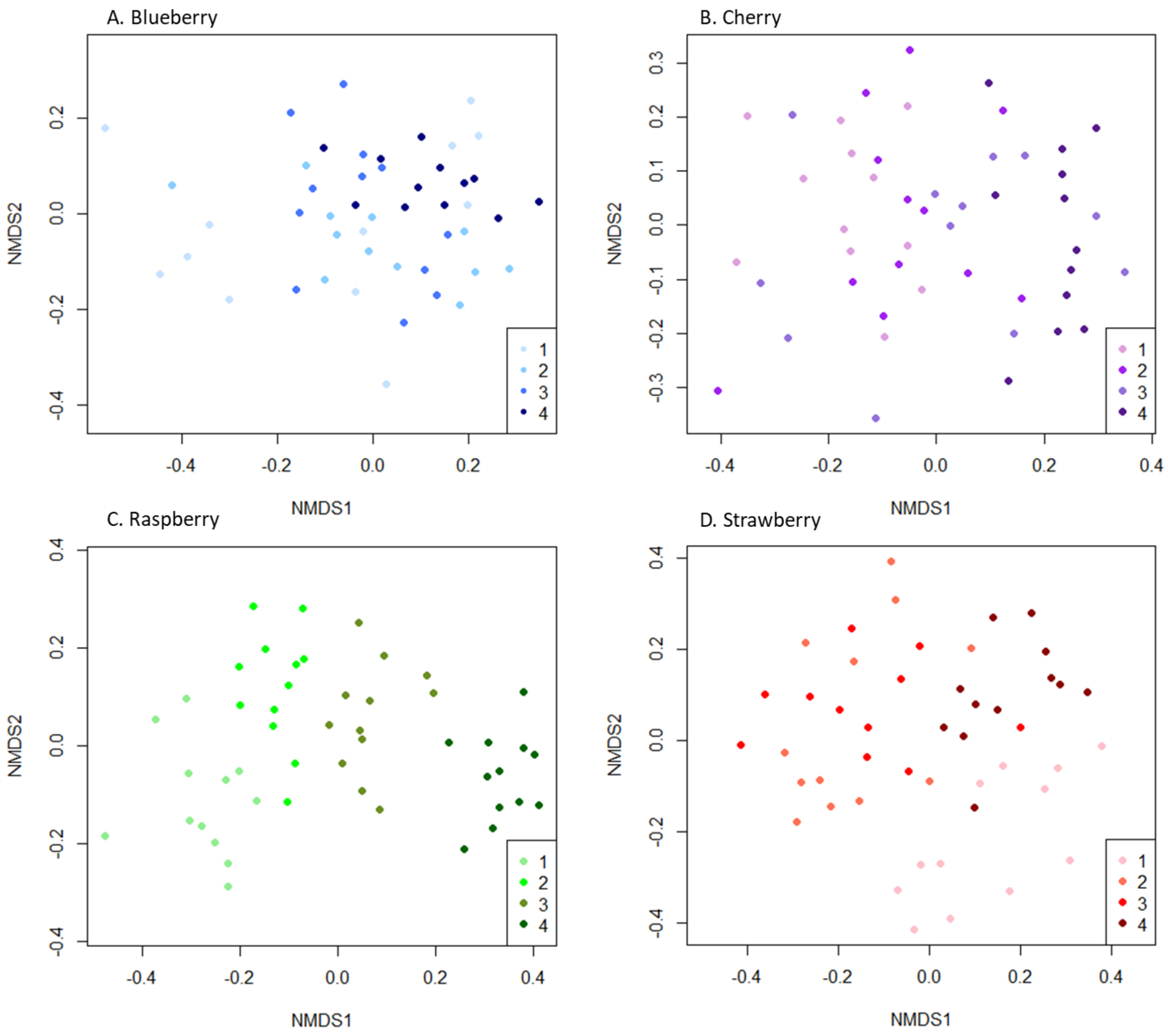

Fig. S6 Four separate Nonmetric Multidimensional Scaling analyses of binary Jaccard measures of community dissimilarity of fungal communities on four ripening stages for four separate fruit species. Panel A. blueberry (blue dots), B. cherry (purple), c. raspberry (green) and strawberry (red). Ripening stages for each fruit, are from green fruit through to harvest fruit and are denoted by shade of fruit colour, lightest shade for green fruit and moving through to darkest shade for harvest). Exact colours for fruit and stage are denoted in the keys (boxes bottom righthand side of each plot).

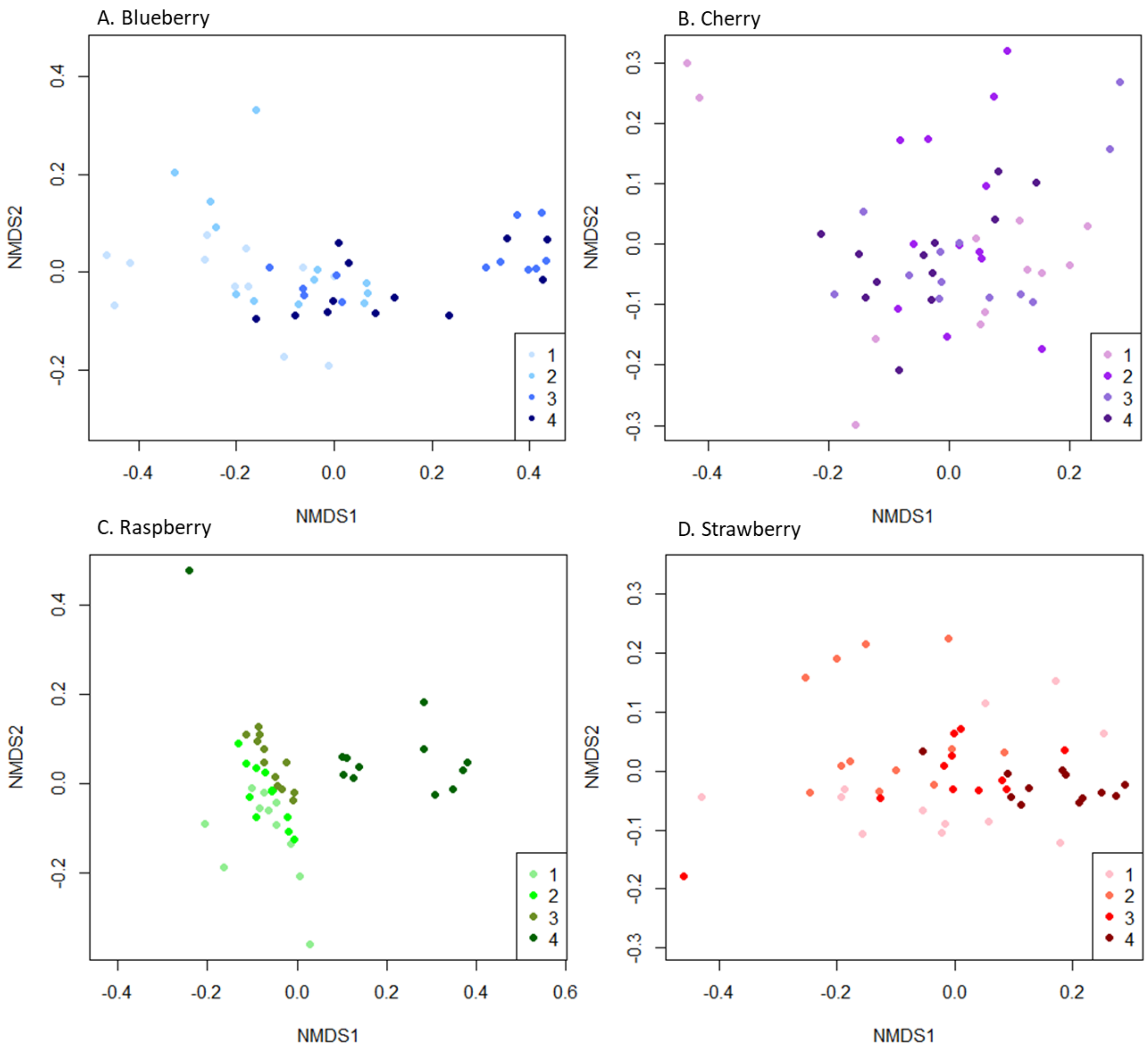

Fig. S7 Four separate Nonmetric Multidimensional Scaling analyses of non-binary Jaccard measures of community dissimilarity of fungal communities on four ripening stages for four separate fruit species. Panel A. blueberry (blue dots), B. cherry (purple), c. raspberry (green) and strawberry (red). Ripening stages for each fruit, are from green fruit through to harvest fruit and are denoted by shade of fruit colour, lightest shade for green fruit and moving through to darkest shade for harvest). Exact colours for fruit and stage are denoted in the keys (boxes bottom righthand side of each plot).

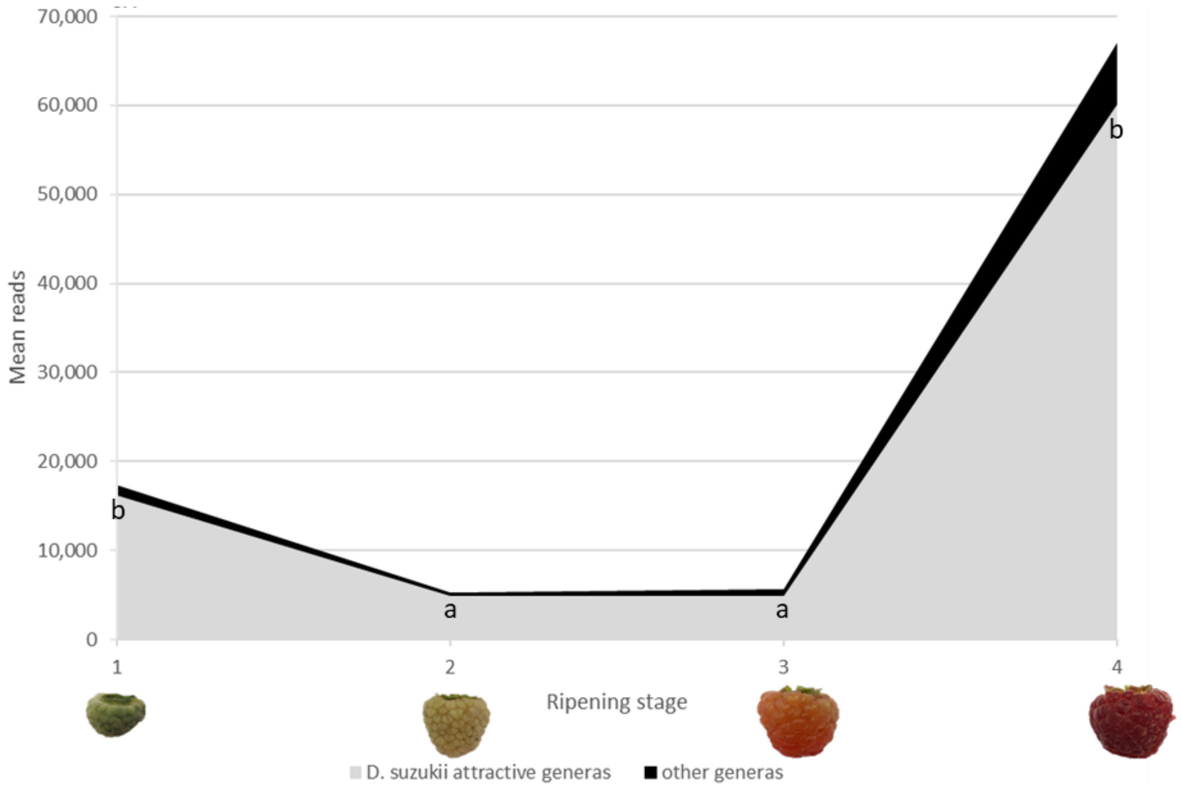

Fig. S8 Relative abundance of known *D. suzukii* attractive yeast genera (*Hanseniaspora, Pichia, Saccharomyces*, *Candida* and *Metschnikowia* (grey) and other yeast genera (black) on ripening raspberries. Ripening stage of raspberries had a significant effect on the abundance of these attractive genera (Kruskal-Wallis chi-squared = 28.70, P = 2.59 x10^{-6}). Different lower-case letters show significant differences in relative abundance of attractive genera between ripening stages at $P < 0.05$, Dunn's comparisons post-hoc with Benjamini–Hochberg multiple comparison correction.

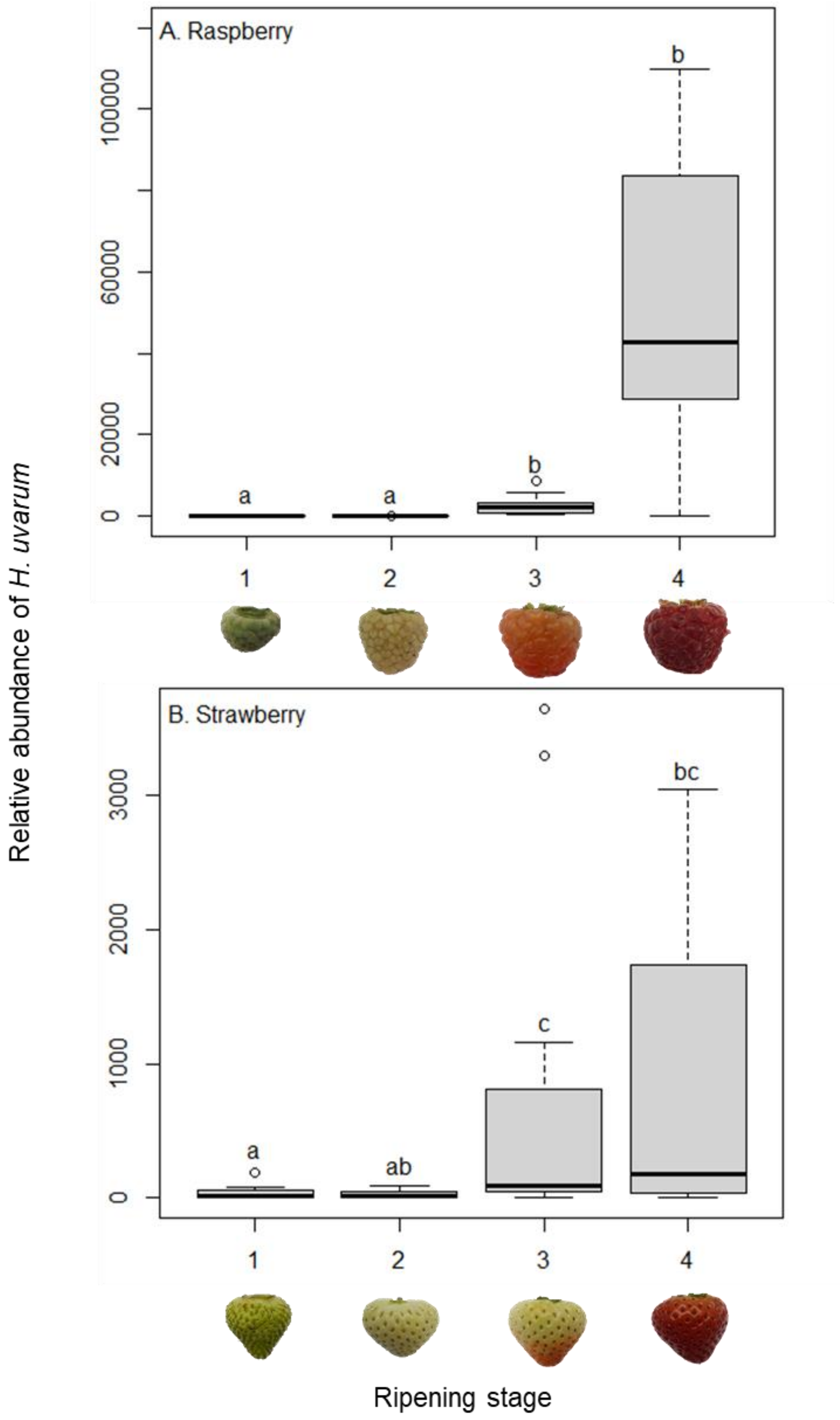

Fig. S9 Relative abundance of *H. uvarum* across ripening stages of A. raspberries and B. strawberries. For these fruit ripening stage had a significant effect on *H. uvarum* relative abundance (Kruskal-Wallis chi-squared = 33.40, df = 3, P = 2.66 x10^{-7}; chi-squared = 12.59, df = 3, P = 0. 0056).

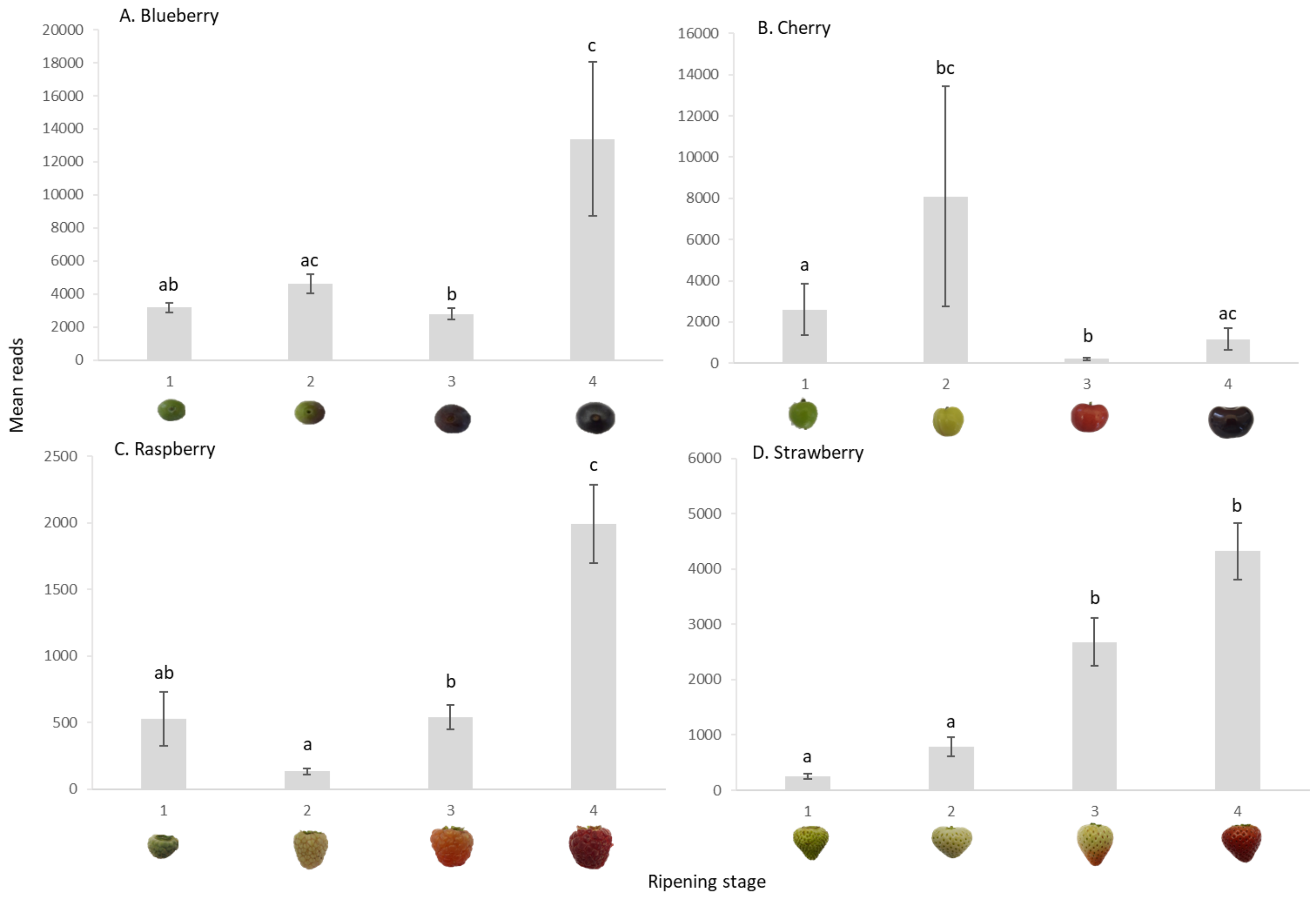

Fig. S10 Relative abundance of *B. cinerea* across fruit type and maturation stages (1, unripe/green fruit; 2, de-greening fruit; 3, ripening fruit; and 4, fully ripe/harvest fruit) for blueberry, cherry, raspberry and strawberry. When fruit types were analysed individually, ripening stage had a significant effect on relative read abundance of *B. cinerea* for all fruits ($P < 0.003$). Different letters show any significant differences between ripening stages within each fruit separately.

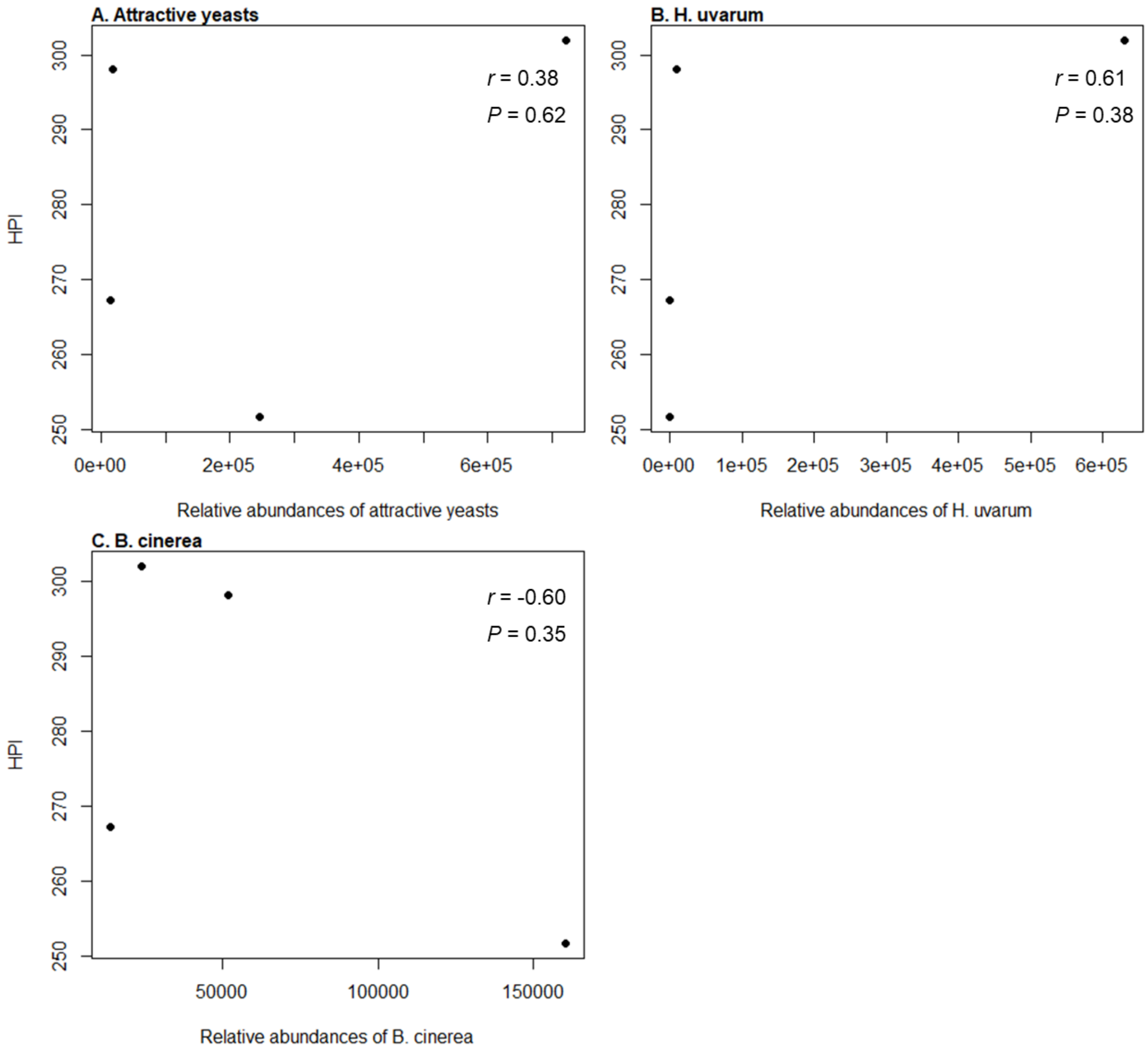

Fig. S11 Scatter plots showing the relationship between HPI values from Bellamy et al.[38] and total reads of (A) attractive Saccharomycetales yeast, (B) *H. uvarum*, and (C) *B. cinerea*. Pearson's correlation statistics, based on four comparisons, are in the top right of each plot.

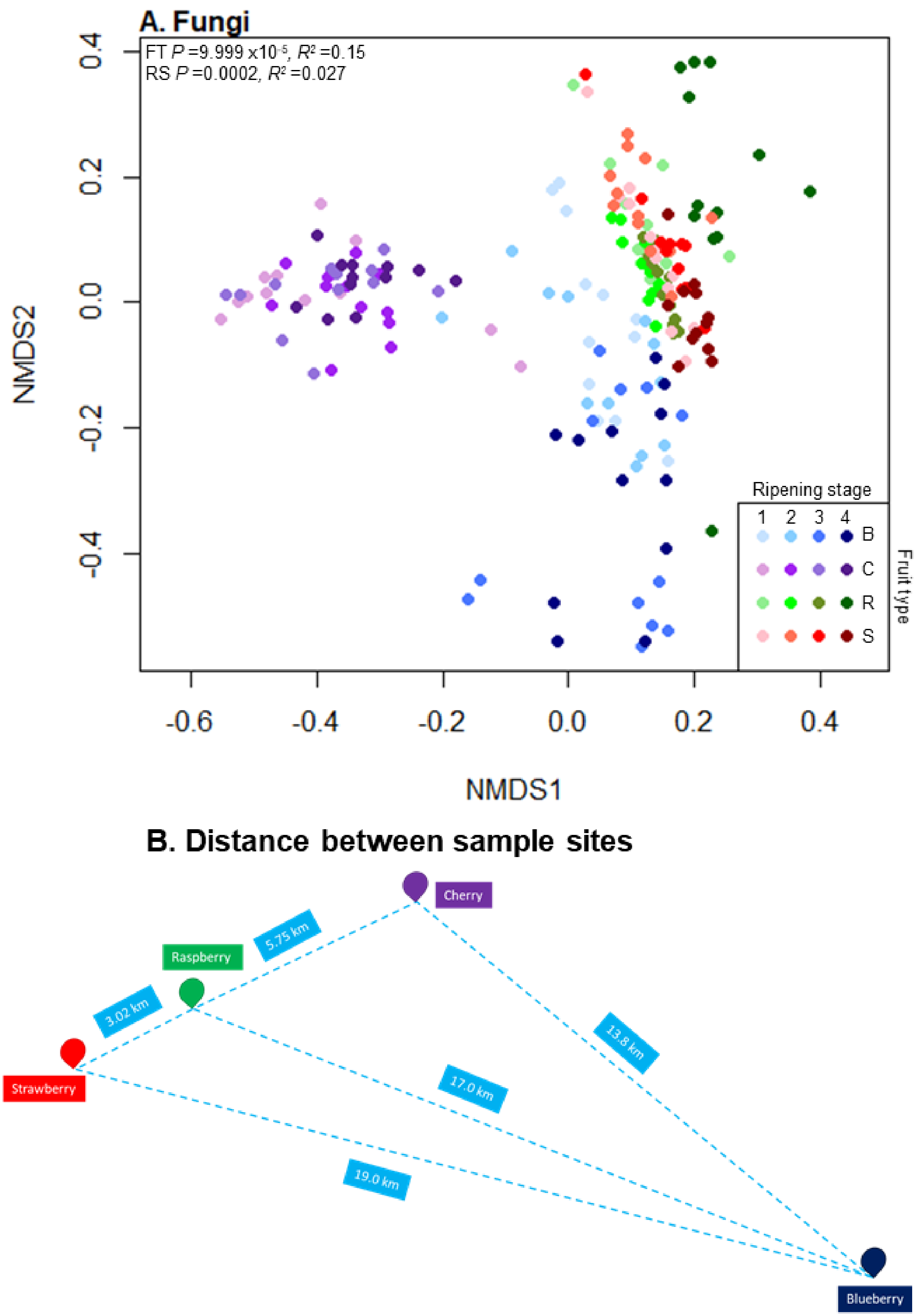

Fig. S12 A. NMDS plots representing the differential abundances of fungal phylotypes. Nonmetric Multidimensional Scaling (NMDS) plots of abundance Jaccard measures of community dissimilarity of total fungal communities (Fig. 4). Fruits were sampled from different geographic locations and B. shows the distance in km between the sample sites.

Table S2. Average Shannon's and Simpson's diversity indexes for the different fruit species and ripening stages.

	Average diversity indexes	
Samples	Shannon	Simpson
Fruit species		
blueberry	1.04 a	0.41 a
cherry	1.41 b	0.58 b
raspberry	1.13 a	0.41 a
strawberry	1.36 b	0.48 c
Ripening stage		
1	1.26 ab	0.034 ab
2	1.29 a	0.044 a
3	1.08 b	0.051 b
4	1.31 a	0.041 a

Table S3. Results from pairwise PermANOVA on binary Jaccard distance matrices for fruit species.

Fruit species		R^2	P
blueberry	cherry	0.09	9.999×10^{-5}
blueberry	raspberry	0.1	9.999×10^{-5}
blueberry	strawberry	0.16	9.999×10^{-5}
cherry	raspberry	0.14	9.999×10^{-5}
cherry	strawberry	0.2	9.999×10^{-5}
raspberry	strawberry	0.09	9.999×10^{-5}

Table S4. Results from pairwise PermANOVA on binary Jaccard distance matrices for ripening stages.

Ripening stage		R^2	P
1	2	0.09	9.999×10^{-5}
1	3	0.1	9.999×10^{-5}
1	4	0.1	9.999×10^{-5}
2	3	0.11	9.999×10^{-5}
2	4	0.11	9.999×10^{-5}
3	4	0.12	9.999×10^{-5}

Table S5. Results from pairwise PermANOVA of Saccharomycetales yeasts on binary Jaccard distance matrices for fruit species.

Fruit species		R^2	P
blueberry	cherry	0.06	9.999 x10^{-5}
blueberry	raspberry	0.15	9.999 x10^{-5}
blueberry	strawberry	0.14	9.999 x10^{-5}
cherry	raspberry	0.14	9.999 x10^{-5}
cherry	strawberry	0.10	9.999 x10^{-5}
raspberry	strawberry	0.13	9.999 x10^{-5}

Table S6. Results from pairwise PermANOVA of Saccharomycetales yeasts on binary Jaccard distance matrices for ripening stages.

Ripening stage		R^2	P
1	2	0.09	9.999 x10^{-5}
1	3	0.10	9.999 x10^{-5}
1	4	0.07	9.999 x10^{-5}
2	3	0.10	9.999 x10^{-5}
2	4	0.08	9.999 x10^{-5}
3	4	0.12	9.999 x10^{-5}

Table S7. Results from pairwise PermANOVA on abundance Jaccard distance matrix for fruit species.

Fruit species		R^2	P
blueberry	cherry	0.43	9.999 x10^{-5}
blueberry	raspberry	0.23	9.999 x10^{-5}
blueberry	strawberry	0.25	9.999 x10^{-5}
cherry	raspberry	0.54	9.999 x10^{-5}
cherry	strawberry	0.57	9.999 x10^{-5}
raspberry	strawberry	0.11	9.999 x10^{-5}

Table S8. Results from pairwise PermANOVA on abundance Jaccard distance matrix for ripening stages.

Ripening stage		R^2	P
1	2	0.15	9.999×10^{-5}
1	3	0.16	9.999×10^{-5}
1	4	0.12	9.999×10^{-5}
2	3	0.21	9.999×10^{-5}
2	4	0.16	9.999×10^{-5}
3	4	0.20	9.999×10^{-5}

Table S9. Results from pairwise PermANOVA of Saccharomycetales yeasts on abundance Jaccard distance matrix for fruit species.

Fruit species		R^2	P
blueberry	cherry	0.05	9.999×10^{-5}
blueberry	raspberry	0.07	9.999×10^{-5}
blueberry	strawberry	0.07	9.999×10^{-5}
cherry	raspberry	0.10	9.999×10^{-5}
cherry	strawberry	0.09	9.999×10^{-5}
raspberry	strawberry	0.09	9.999×10^{-5}

Table S10. Results from pairwise PermANOVA of Saccharomycetales yeasts on abundance Jaccard distance matrix for ripening stages.

Ripening stage		R^2	P
1	2	0.045	9.999×10^{-5}
1	3	0.047	9.999×10^{-5}
1	4	0.038	9.999×10^{-5}
2	3	0.047	9.999×10^{-5}
2	4	0.040	9.999×10^{-5}
3	4	0.068	9.999×10^{-5}

Table S14: Sampling dates for the four fruits (cherry, blueberry, raspberry, and strawberry) at the four ripening stages (from unripe/green fruit to fully ripe/harvest fruit).

Fruit species	ripening stage		sampling date
cherry	1	green	13.6.18
	2	white/pink	28.6.18
	3	red	29.6.18
	4	purple (fully ripe/harvest)	3.7.18
blueberry	1	green	27.6.18
	2	green/purple	9.7.18
	3	purple	12.7.18
	4	navy (fully ripe/harvest)	18.7.18
raspberry	1	green	16.7.18
	2	white	3.8.18
	3	pink	14.8.18
	4	red (fully ripe/harvest)	31.8.18
strawberry	1	green	3.8.18
	2	white	14.8.18
	3	pink	31.8.18
	4	red (fully ripe/harvest)	11.9.18
